# NADPH Dehydrogenase Gene Regulates Energy Distribution and Fatty Acid Metabolism During Fruiting Body Formation in the Filamentous Fungus *Podospora anserina*

**DOI:** 10.1111/1751-7915.70401

**Published:** 2026-06-15

**Authors:** Yanling Qiu, Amechi S. Nwankwegu, Yue Sun, Yuanjing Li, Weizhao Chen, Gang Liu, Ning Xie

**Affiliations:** ^1^ Shenzhen Key Laboratory of Microbial Genetic Engineering, College of Life Sciences and Oceanography Shenzhen University Shenzhen People's Republic of China; ^2^ Department of Rheumatology and Immunology, Shenshan Medical Center, Sun Yat‐Sen Memorial Hospital Sun Yat‐Sen University Shanwei China

**Keywords:** cellular respiration, fatty acid metabolism, gene deletion, NADPH dehydrogenase, *Podospora anserina*

## Abstract

Nicotinamide adenine dinucleotide phosphate (NADPH) dehydrogenase is an oxidoreductase involved in many physiological processes and metabolic pathways. However, its role in filamentous fungal physiology is still unclear. In the present study, three canonical NADPH dehydrogenase genes (*Panph1*, *Panph2,* and *Panph3*) in the fungus *Podospora anserina* were deleted, and multiple mutants were constructed. Results show a significantly increased number of fruiting bodies in the NADPH dehydrogenase mutant, with the *nph* ^*ΔΔΔ*^ triple mutant exhibiting higher sensitivity to oxidative stress, suggesting an active‐site protein misfolding. Specifically, the antioxidant genes Nox and CAT in the WT were significantly down‐regulated, confirming NADPH availability; however, in the NADPH mutant, these genes were significantly upregulated (*p* ≤ 0.001) as a response to nullify the constraint imposed by NADPH deletion and alleviate oxidative stress. Furthermore, an increase in substrate‐level phosphorylation compensated for a significant decrease in oxidative phosphorylation due to NADPH gene deletion. The NADPH/NADP^+^ ratio, a driving force for the intracellular redox potential, showed a significant increase in the *nph* ^*ΔΔΔ*^ triple mutant. Meanwhile, the NADPH mutant inhibited the *β*‐oxidative pathway, decreasing fatty acid degradation, but promoted fatty acid biosynthesis, reflecting the role of NADPH in the metabolic programming of cellular respiration and energy utilization processes in fungi. Our study provides genetic evidence for the role of the NADPH dehydrogenase gene in oxidative defence and energy metabolism in *P. anserina* .

## Graphical abstract

NADPH dehydrogenase gene deletion significantly triggered NADPH/NADP^+^ increase in mutant strains. NADPH gene deletion promoted Fatty acid biosynthesis and repressed *β*‐oxidation. NADPH gene deletion decreased oxidative phosphorylation and increased substrate‐level phosphorylation. Mutants exhibited hypersensitivity to oxidative stress, but promoted more fruiting body differentiations.

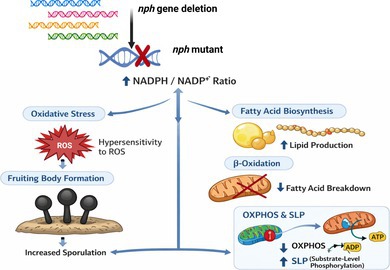

## 1 Introduction

The fungal fruiting body is a main physiological feature that differentiates complex multicellular structures during sexual development and ultimately facilitates spore production and dispersal (Nwankwegu et al. 2025). As a biomarker of reproductive growth, fruiting body development represents the transformation of fungal growth forms, driven by exogenous and endogenous stimulations. In filamentous fungi, NADH dehydrogenase, as an enzyme, coordinates hyphal differentiation, sexual reproduction, and fruiting body formation, linking cellular respiration with morphogenesis, reproductive fitness, and adaptation to environmental conditions. Environmental changes are exogenous stimuli, which are triggered by temperature drawdown, nutrient depletion, and shifts in light exposure, whereas endogenous stimuli are mainly triggered by abnormal expression of structural and regulatory genes in microorganisms (Lalucque et al. 2017), which in turn induce abnormal protein conformations.

Nicotinamide adenine dinucleotide phosphate (NADPH) dehydrogenase is a flavoprotein that uses the cofactors flavin adenine dinucleotide (FAD) and flavin mononucleotide (FMN) and belongs to the family of oxidoreductases, widely distributed across diverse plants and microorganisms (Ding et al. 2024). NADPH dehydrogenase is involved in oxidative phosphorylation by oxidizing NADPH to NADP^+^ to transfer electrons to the respiratory chain (Koju et al. 2022). The NADPH dehydrogenase exhibits functional diversity, including roles in cellular homeostasis, signal transduction, and regulation of ROS signalling (Mondal et al. 2025). In fungi, NADPH‐dependent oxidoreductases and NADPH oxidases are increasingly recognized as central regulators of developmental signalling because they couple cellular reducing power to the generation of reactive oxygen species, mitochondrial metabolism, and anabolic biosynthesis. It also participates in a wide range of physiological and biochemical processes, such as biosynthesis and the TCA cycle, thereby regulating growth and development in organisms (Chen et al. 2024).

The filamentous fungus *Podospora anserina* is an important model for investigating the molecular regulation of fungal development, mitochondrial metabolism, and aging, given its ease of molecular genetic manipulation, short one‐week life cycle, and availability of easily quantifiable aging characteristics (Ament‐Velásquez and Vogan 2022; Hamann and Osiewacz 2022; Warnsmann et al. 2021). Fruiting body development in this ascomycete is an energetically demanding developmental process, and usually requires coordinated regulation of nutrient allocation, respiration, and redox homeostasis. Previous studies demonstrated that NADPH oxidase‐associated pathways are key to fungal differentiation and developmental signalling. Specifically, disruption of the PaNox1 NADPH oxidase in *P. anserina* has been found to impair fruiting body differentiation, while PaNox2 controls ascospore germination (Malagnac et al. 2004), highlighting the developmental significance of NADPH‐dependent redox systems. Furthermore, ROS‐mediated signalling downstream of NADPH oxidases has been linked to MAP kinase cascades, stationary‐phase differentiation, and multicellular perithecial maturity (Lalucque et al. 2017). Studies on respiratory‐deficient mutants of *P. anserina* demonstrated strong metabolic reprogramming, including induction of the alternative oxidase, activation of glycolysis, and altered lifespan regulation, suggesting a tight coupling between mitochondrial energy distribution and cellular metabolism (Humbert et al. 2015). Additionally, recent evidence further indicates that developmental regulation in filamentous fungi is closely linked to metabolic rewiring, particularly fatty acid catabolism and mitochondrial energy distribution (Li et al. 2026). During sexual development, fungi require substantial lipid mobilization to support membrane biogenesis, multicellular tissue formation, and ascospore maturation and dispersal (Niu et al. 2020). However, despite increasing evidence for NADPH‐dependent signalling in fungal development, the specific role of NADPH dehydrogenase genes in coordinating fatty acid metabolism and energy allocation during fruiting body formation remains poorly understood. Therefore, investigating NADPH dehydrogenase function in *P. anserina* may provide important mechanistic insight into how mitochondrial redox metabolism coordinates fungal development, differentiation, and reproductive success. Consequently, we characterized three genes encoding NADPH dehydrogenase in the filamentous fungus and evaluated their functional roles in the central metabolic pathway and fatty acid metabolism. Further, phenotypic characteristics, including mycelial growth, sexual reproduction (e.g., fruiting body formation), oxidative stress, and hyphal reduction, were also assessed. The study offers crucial insights into NADPH dehydrogenase activity during fungal fruiting body development.

## 2 Materials and Methods

### 2.1 Strains and Culture Conditions

All *P. anserina* strains used throughout this study were derived from the “S” wild‐type (WT) strain, ensuring a homogenous genetic background (Espagne et al. 2008). The *mus51* ^*Δ*^ *::*hygroR and *mus51* ^*Δ*^ *::*nourseoR mutant strains differed from the WT strain by a single deletion of the *mus‐51* gene, effectively increasing the frequency of targeted gene replacement (El‐Khoury et al. 2008). The most recent protocols for standard culture conditions, media, gene sequences, and genetic approaches for *P. anserina* have been previously described and are accessible at http://podospora.i2bc.paris‐saclay.fr/.

### 2.2 Phylogenetic Analysis

Three NADPH dehydrogenase genes of *P. anserina, Pa_1_9760*, *Pa_4_60*, and *Pa_6_6330*, hereafter (*Panph1*, *Panph2*, and *Panph3*, Table S1), hereafter referred to as *nph*, were obtained from the gene database. A total of 14 NADPH dehydrogenase amino acid sequences from various fungi, plants, bacteria, and protists were available from the genomic databases, including SMZ56269 ( *Serratia grimesii* ), EAU89465 (*Coprinopsis cinereal*), EPE07180 (*Ophiostoma piceae*), KHE80735 (*Neurospora crassa*), EPE08229 (*Ophiostoma piceae*), GAT28090 (*Aspergillus luchuensis*), CAQ43530 (*Zygosaccharomyces rouxii*), CAQ43373 (*Zygosaccharomyces rouxii*), EDN60974 ( *Saccharomyces cerevisiae* ), ABN67047 (*Scheffersomyces stipites*), BAB76910 (*Nostoc* sp.), EAA22988 (*Plasmodium yoelii*), CAB79624 ( *Arabidopsis thaliana* ), and AJ245862 ( *Solanum tuberosum* ). Deduced protein sequences were aligned using MAFFT and adjusted manually by MEGA7 (Kumar et al. 2016). Maximum likelihood analysis was performed in RaxMLGUI v. 1.3 (Silvestro and Michalak 2012). The optimal ML search was conducted over 1000 generations under the GTR + GAMMA substitution model. The phylogenetic tree was reviewed in TreeViewer (Bianchini and Sánchez‐Baracaldo 2024). The GTR + GAMMA is selected for protein sequence analysis because it captures complex substitution dynamics, improves phylogenetic reliability, and accounts for evolutionary rate variation.

### 2.3 NADPH Dehydrogenases Gene Deletion

Deletion cassettes for *nph1*, *nph2*, and *nph3* were constructed using the split‐marker method, as previously described (Xie et al. 2015). In the case of *nph1*, a geneticin resistance cassette was used (Chan Ho Tong et al. 2014), while a phleomycin resistance cassette was used for *nph2*, and a hygromycin B resistance cassette for *nph3* (Tangthirasunun et al. 2017). The resulting PCR products with geneticin, phleomycin, or hygromycin cassettes transformed the *mus51::nourseoR protoplast*s. Transformants were selected on a medium containing 100 μg/mL Geneticin, 20 μg/mL Phleomycin, or 75 μg/mL Hygromycin B. Putative deleted mutants were first selected by PCR. The PCR amplified specific junctions to the replaced locus using two primers that annealed at one end of the selectable resistance gene and upstream of the proximal flank used in the deletion cassette (Table S2). Homologous recombination of the deletion cassette enabled amplification of a predictable fragment on either side of the selectable resistance gene. Two or three candidate transformants were genetically purified by crossing them to the WT strain to eliminate potential untransformed nuclei and segregate the ^*Δ*^ *mus‐51* mutation. Several mat^+^ and mat^−^ single mutants of *nph1* ^*Δ*^, *nph2* ^*Δ*^, and *nph3* ^*Δ*^ lacking *mus‐51* were selected from each progeny and subjected to Southern blot analysis for final validation (Figure S1). The nucleic acid extraction and manipulation methods have been previously described (Bouex et al. 2002). Purified transformants verified by Southern blot were chosen as stock deletion mutants for subsequent studies. The double *nph1* ^*Δ*^ *nph2* ^*Δ*^, *nph1* ^*Δ*^ *nph3* ^*Δ*^, and *nph2* ^*Δ*^ *nph3* ^*Δ*^, as well as the triple *nph* ^*ΔΔΔ*^ mutants, were constructed by genetic crosses, and relevant genotypes were confirmed by PCR (Xie et al. 2018).

### 2.4 Enzyme Activity Assay

To detect NADPH dehydrogenase activity and NADP(H) concentration in *P. anserina*, WT and mutant strains were cultured for 2 days at 27°C on M2 medium containing cellophane. The grown hyphae from two plates were harvested, placed in centrifuge tubes containing 0.1 M Tris–HCl buffer (pH 7.8), and broken by sonication. The supernatant was centrifuged at 10,000 rpm for 5 min, and the pellet was separated. The NADPHD, NADPH, and NADP kits (Shanghai Enzyme‐linked Biotechnology Co. Ltd) were used to determine NADPH dehydrogenase activity in the WT and mutant strains. Furthermore, the oxidation of cytosolic NADP, NADPH, and the NADPH/NADP^+^ ratio was estimated accordingly.

### 2.5 Phenotypic Analyses

To determine the roles of *nph1*, *nph2*, and *nph3* genes in fungal development and physiology, the mutants and the WT strain were incubated separately on M_2_ medium at 27°C. Colony morphology, pigmentation, perithecium formation, ascospore production, dispersal, and germination were observed during their vegetative growth and sexual cycle. Growth phenotypes were recorded after 3, 5, and 7 days, and ImageJ was used to quantify the fruiting body production. About 200 spores from each plate were picked randomly and transferred to the germination culture medium. After culturing for 24 h in the dark, the number of germinated spores was counted, and the relative spore germination rate was calculated from triplicate measurements.

### 2.6 Sensitivity to Oxidative Stress Assay

To ascertain whether NADPH dehydrogenase of *P. anserina* has a defensive function against ROS, thereby reducing oxidative damage, the relative sensitivity of the strains to H_2_O_2_ was evaluated. WT and mutant (*nph* ^*ΔΔΔ*^) were inoculated on M_2_ plates supplemented with 200 μL of H_2_O_2_ at varying concentrations: 0.025%, 0.05%, and 0.1%. Following these, relative growth and fertility were estimated each day.

### 2.7 Assessment of Growth in Fatty Acids

*P. anserina* usually grows on an M_2_ medium containing dextrin as the sole carbon source in the lab. To test the mutants' ability to metabolize fatty acids, their growth on a medium containing fatty acids as the sole carbon source was assessed (Scheckhuber and Osiewacz 2008). The minimal synthetic medium was used with fatty acids as a carbon source: 1% (V/V) Tween20, 1% (V/V) Tween40, 1% (V/V) Tween80, 20 mM hexanoic acid (C6), 6 mM oleic acid (C18:1), C6 and C18, and C18:1. These were solubilized with 0.2% (V/V) Tween40. The shattered mycelia were inoculated and grew parallel to one another on plates containing different carbon sources (Tang et al. 2008).

### 2.8 Analysis of Gene Expressions by RT qPCR

Quantitative real‐time PCR was used for gene expression analysis (Jozefczuk and Adjaye 2011). The WT and the triple mutant *nph* ^*ΔΔΔ*^ were grown in a liquid medium at 27°C for 3 days. The 100 μL lysed mycelia were added to 100 mL of liquid medium. Total RNA was isolated using the RNeasy Plant Mini Kit (Qiagen). RNA integrity was monitored by measuring optical density and agarose gel electrophoresis. Complementary DNA (cDNA) was synthesized from 1 μg of total RNA using the PrimeScript RT reagent Kit with a gDNA eraser (TaKaRa, Japan). Real‐time PCR measurements were performed using the TB Green PremixTM Ex TaqTM II (Tli RNaseH Plus) (TaKaRa, Japan) and detection on a qTOWER^3^ (Analytik Jena AG, Germany). Experimental real‐time amplification and reaction conditions, including normalization, are detailed in Table S3. The protodermal factor 2 (PDF2), as the protein phosphatase (PP2A) regulatory subunit A (Pa_7_6690) gene, was used as the normalization reference gene.

### 2.9 Statistical Analysis

Data represent the mean of three replicates (*n* = 3), and statistical analysis for all comparative analyses, including enzymatic activity, phenotypic characteristics, gene expression, and fatty acid metabolism, was performed using a *t*‐test in GraphPad and Origin software (2021). Error bars represent standard deviation (±SD).

## 3 Results

### 3.1 Identification of NADPH Dehydrogenase Genes and Phylogenetic Analysis

**FIGURE 1:**
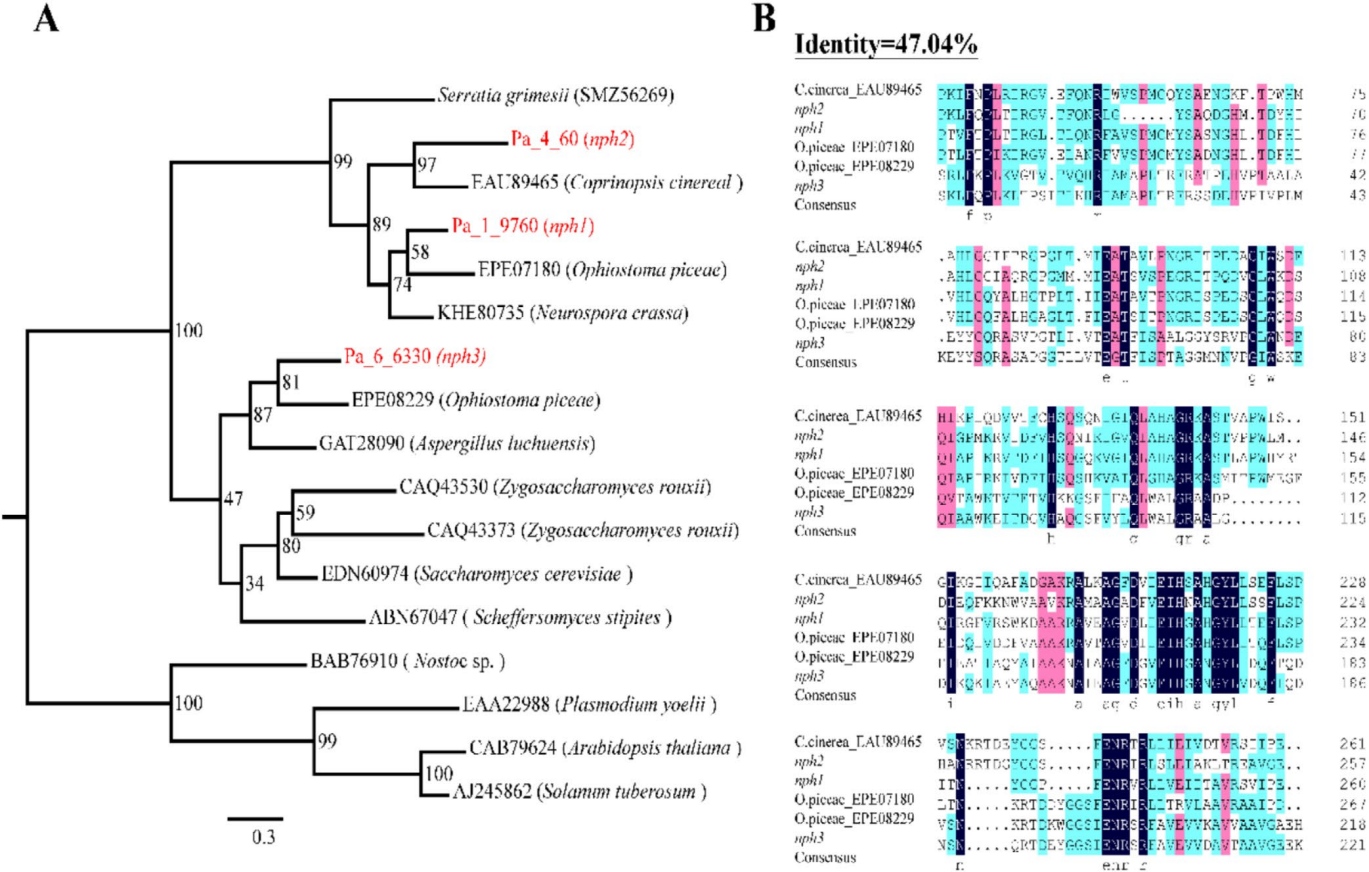
Identification of NADPH dehydrogenase in *P. anserina* . (A) Phylogenetic analysis including NADPH dehydrogenase‐like protein amino acid sequences of plants, fungi, bacteria, and protists. The red genes highlighted were the putative NADPH dehydrogenase genes in *P. anserina* . Deduced protein sequences were aligned using MAFFT and adjusted manually by MEGA7.0. Maximum likelihood analysis was performed in RaxMLGUI v. 1.3. The optimal ML search was conducted with 1000 generations under the GTR + GAMMA substitution model evolution. The phylogenetic tree was reviewed in TreeView. The distance scale is shown at the bottom left of the figure. As *P. anserina* is an ascomycete, representative sequences from major fungal lineages in the phylum Ascomycota (*Ophiostoma piceae*) and Basidiomycota (*Coprinopsis cinerea*) were selected to capture common evolutionary spectra and divergence, respectively. Accordingly, the rationale for inclusion is reflected in their similar habitat colonization, fast growth, saprophytic nature, stress tolerance, and environmental sensing and plasticity. Specifically, like *P. anserina*, these fungi are filamentous and thrive on lignocellulosic substrates, including wood, cow dung, decaying organic matter, and soil, suggesting similar ecological functions. Further, homologues from *P. Yoeltii, A. thaliana*, and *S. tuberosum* were included as outgroups to root the phylogenetic tree. (B) The overall amino acid sequence identity among three NADPH dehydrogenase proteins and the three closest protein sequences in evolutionary trees (*nph1*, *nph2*, *nph3*, EPE07180_*O. piceae*, EAU89465_*C. cinerea*, and EPE08229_*O. piceae*). Fusia represents the amino acid sequence with a homology level ≥ 75%, and conserved amino acids with a homology level ≥ 50% are shown in turquoise. The homologous parts are highlighted in blue.

The three putative NADPH dehydrogenase amino acid sequences were obtained from the genomic databases of *P. anserina*, including *Pa_1_9760* (*Panph1*), *Pa_4_60* (*Panph2*), and *Pa_6_6330* (*Panph3*). Fourteen NADPH dehydrogenase protein sequences retrieved from NCBI were aligned with *nph1*, *nph2*, and *Pnph3* using MAFFT, and the alignments were manually adjusted in MEGA7.0. A phylogenetic tree was constructed using the Maximum likelihood method (Figure 1A). The evolutionary relationship among groups is shown intuitively. Three proteins with the highest similarity to NADPH dehydrogenases in *P. anserina* were EPE07180 (*Ophiostoma piceae*), EAU89465 (*Coprinopsis cinerea*), and EPE08229 (*Ophiostoma piceae*). The *nph1*, *nph2*, and *nph3* showed 59%, 51%, and 55% similarity at the amino acid sequence level to EPE07180 (*Ophiostoma piceae*), XP_001832304 ( *C. cinerea* ), and EPE08229 (*O. piceae*), respectively. Other homologues, including EAA22988 (*Plasmodium yoelii*), CAB79624 ( *A. thaliana* ), and AJ245862 ( *Solanum tuberosum* ), showed much lower sequence similarity, indicating that the NADPH dehydrogenase of *P. anserina* considerably differed from them. Additionally, the identity analysis of NADPH dehydrogenase suggested that the overall percentage of amino acid sequence identity among the three *nph* proteins and three closest protein sequences (EPE07180, EAU89465, and EPE08229) is 47.04% (Figure 1B), suggesting that *nph1*, *nph2*, and *nph3* are homologues of the NADPH dehydrogenase gene, thus are duplicated genes within the *P. anserina* genome and may evolve specialized or divergent functions as paralogs.

### 3.2 Effect of NADPH Gene Deletion on Enzyme Activity and Fruiting Body Differentiation

**FIGURE 2:**
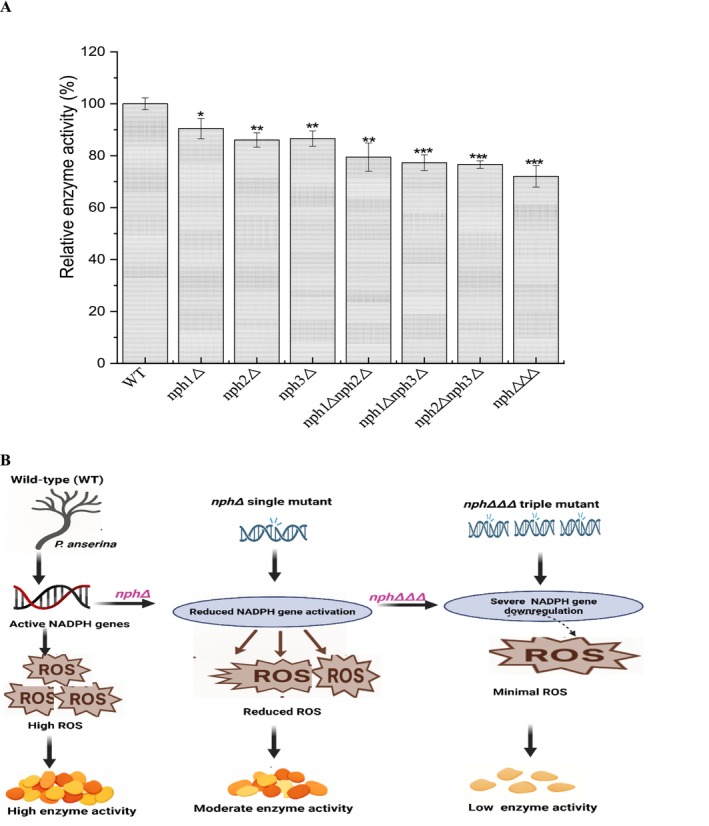
(A) Relative enzyme activities of NADPH dehydrogenase in WT and mutants. The mycelia cultures incubated on M_2_ medium for 2 days were collected and smashed in 0.1 M Tris–HCl buffer, pH 7.8. After centrifugation, NADPH dehydrogenase activities of mutants and wild‐type (WT) were assayed by the NADPHD kit. Activity is expressed relative to the WT strain and is considered 100%. Data are a mean of three replicates, and error bars shown are standard deviations. *t*‐test *p ≤ 0.05, **p ≤ 0.01, ***p ≤ 0.001. (B) Illustrative interactions demonstrating the mechanisms regulating significantly decreased enzyme activity in the triple *nph∆∆∆* mutants relative to the WT and single *nph∆* mutant. The functional NADPH genes in the WT produced ROS, activating enzyme gene expression, and leading to high enzyme production. In single mutants, a partial loss of the NADPH gene reduced ROS but partially activated some enzyme genes, resulting in moderately reduced enzyme activity. Conversely, all NADPH genes in the triple mutants are knocked out, leading to minimal ROS signalling, severe down‐regulation of enzyme gene expression, and a dramatic reduction in enzyme activity. Although direct ROS level assessment was not considered at this point, differential enzyme activities may provide an indirect link between ROS levels in WT and mutant strains. Therefore, the inferred model would benefit from experimental validation of ROS levels. NPH = NADPH dehydrogenase.

An enzyme assay was performed to determine whether the *nph1*, *nph2*, and *nph3* mutants displayed NADPH dehydrogenase activity. Results demonstrated significant decreases in NADPH dehydrogenase activity in the NADPH dehydrogenase gene‐deficient mutants. This is especially true for both the double and triple mutants, retaining ≤ 72% of NADPH dehydrogenase activity (Figure 2A). The average activity of NADPH dehydrogenase in the WT strains was 19.37 ± 0.58 U/L at 37°C. The single mutants showed a slight decrease in enzyme activity, retaining 92% (*nph1* ^*Δ*^), 86% (*nph2* ^*Δ*^), and 90% (*nph3* ^*Δ*^), indicating minimal protein misfolding and functional redundancy among the NADPH dehydrogenase genes. However, a decrease in NADPH dehydrogenase activity in the multiple mutants revealed a strong linear association between enzyme activity and the multiple mutant combinations. In other words, a progressive decrease in NADPH dehydrogenase activity was observed as the number of mutant combinations increased from double to triple, reflecting additive effects. These also suggest that *the nph1, nph2, and nph3* genes, despite their inactivation, could still exert considerable NADPH dehydrogenase activity. However, impacts became significant across multiple mutant combinations, hinting at potential cumulative effects as they acted in concert to drive a significant loss of enzyme activity (Figure 2B).

**FIGURE 3:**
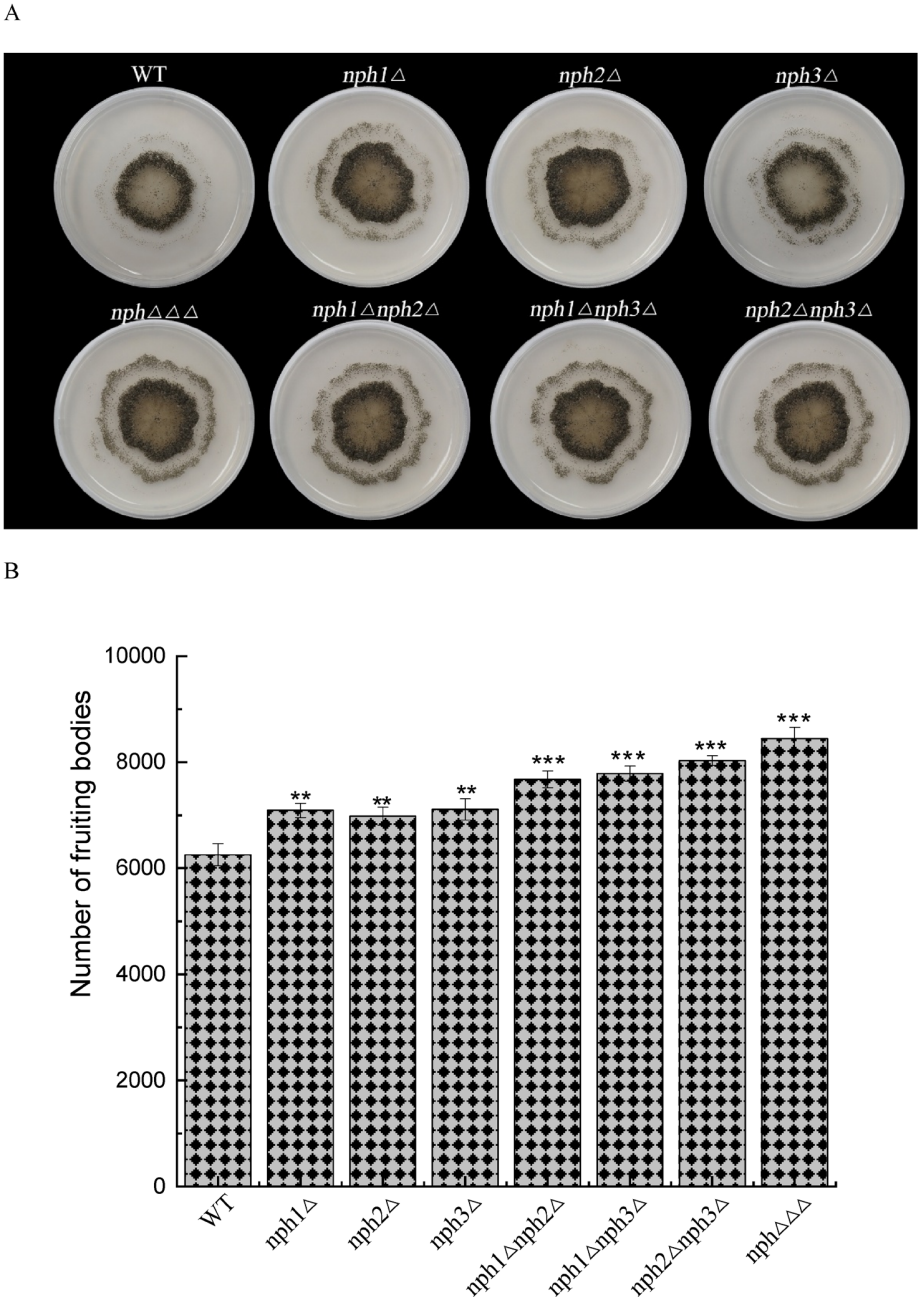
Relative fruiting body formation characteristics between WT and mutant strains. (A) The WT, single (*nph* ^*Δ*^), double (*nph* ^*ΔΔ*^), and triple *nph* ^*ΔΔΔ*^ mutants were grown on M_2_ medium for 7 days. Fruiting bodies were visible as small black dots. The wild‐type strain produced the usual ring of mature perithecia in the centre of the Petri dish. There were two rings of perithecia; the mutants produced more fruiting bodies in the outside ring, which was especially profound in the triple mutant *nph* ^*ΔΔΔ*^. (B) Relative fruiting body numbers of wild‐type and mutants. Mature fruiting bodies produced by mutants and WT on M_2_ media were counted after 7 days of incubation. Data are a mean of three replicates, and error bars shown are standard deviations. *t*‐test *p ≤ 0.05, **p ≤ 0.01, ***p ≤ 0.001.

Further, whether *nph1*, *nph2*, and *nph3* were involved in the sexual development of *P. anserina* was assessed by incubating the mutants and WT strains in M2 minimal medium at 27°C under light, which is the optimal condition for growth and sexual reproduction. After 3 days of incubation, no significant difference in vegetative growth was observed between the mutants and the WT. However, after 7 days of incubation, all mutants produced significantly more fruiting bodies than the WT (Figure 3A), suggesting a potential shift in resource allocation that prioritizes sexual reproduction over vegetative growth. Consequently, the number of fruiting bodies in the single mutants *nph1* ^*Δ*^, *nph2* ^*Δ*^, and *nph3* ^*Δ*^ increased by 14%, 12%, and 14%, respectively, relative to WT. Again, the double mutants *nph1* ^*Δ*^ *nph2* ^*Δ*^, *nph1* ^*Δ*^ *nph3* ^*Δ*^, and *nph2* ^*Δ*^ *nph3* ^*Δ*^ increased by 23%, 24%, and 28%, respectively, while a maximum increase (35%) in the number of fruiting bodies was reported in the triple mutant (Figure 3B). This demonstrates inverse interactions with the enzyme activity above and suggests possible developmental and reproductive trade‐offs allowing the marginal decrease in NADPH dehydrogenase activity to stimulate progressive increases in the sexual reproductive ability (fruiting bodies) in the mutants. Again, a more plausible reason for the stimulated progressive fruiting body development in nph mutants after 7 days could be that they lack ROS‐mediated suppression and senescence acceleration, which would typically limit reproductive structure formation in the WT, promoting a gradual and/or extended reproductive phase with increased perithecial output over time in the mutants. Following significant fruiting body promotion in the triple mutant, subsequent comparisons prioritized WT and *nph* ^*ΔΔΔ*^.

### 3.3 Relative Sensitivity to Oxidative Stress and Gene Expressions

**FIGURE 4:**
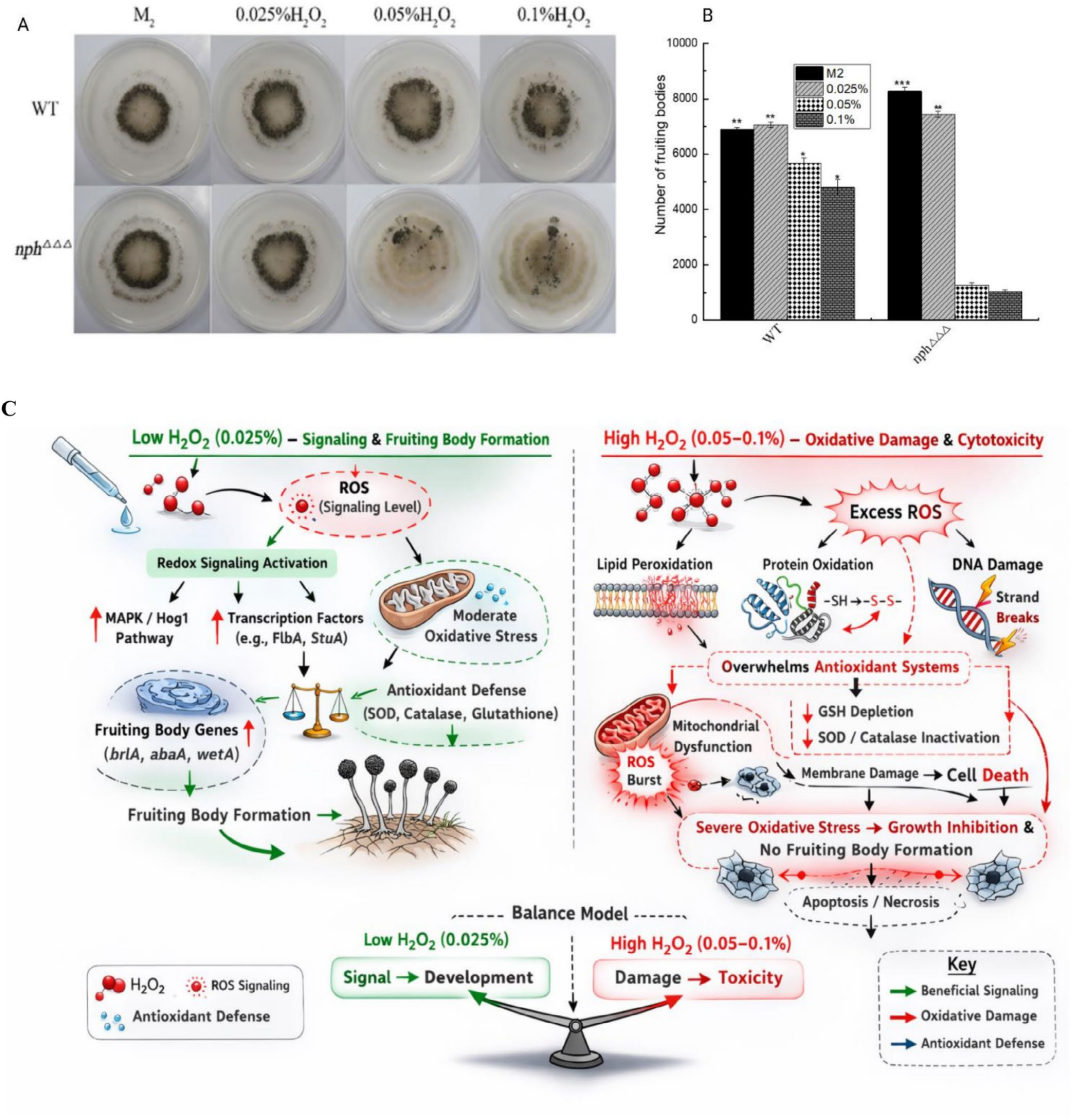
Response of WT and NADPH mutant strains to oxidative stress. (A) Sensitivity to different H_2_O_2_ concentrations. (B) Fruiting bodies were visible as small black dots. Images of wild‐type (WT) and triple mutant *nph* ^*ΔΔΔ*^ were recorded after 7 days of incubation on media spiked with 0, 0.025%, 0.05%, or 0.1% H_2_O_2_. Relative fruiting bodies number of WT and mutants under the chemically modified biotopes. Data are a mean of three replicates, and error bars shown are standard deviations. *t*‐test *p ≤ 0.05, ****p ≤ 0.001. (C) Differential effects of H_2_O_2_ concentrations on *P. anserina* triple *nph∆* mutants—promoting fruiting body formation at low levels (0.025%) but causing severe oxidative damage at higher levels (0.05%–0.1%), indicating the dual role of reactive oxygen species (ROS) as both signalling molecules (co‐substrate) and cytotoxic agents/oxidative damage‐inducers.

Understanding how NADPH gene inactivation impacts oxidative damage in *P. anserina* is crucial; thus, we investigated whether NADPH dehydrogenase could counteract potential ROS‐induced damage, such as free radical intermediate toxicity. We monitored the growth of WT and NADPH dehydrogenase gene mutants on H_2_O_2_‐spiked medium at varying concentrations. The result showed concentration‐dependent variable outcomes in fruiting bodies formation between the WT and mutant strains (Figure 4A). On M_2_ without H_2_O_2_ amendment, a significant (*p* ≤ 0.001, 85%) number of fruiting bodies was observed in the *nph* ^*ΔΔΔ*^ relative to WT (69%, Figure 4B), indicating the ability of the NADPH dehydrogenase mutant to potentially out‐compete the WT in a typical stress‐free condition. On M_2_ supplemented with 0.025% H_2_O_2_, the fruiting body number in *nph* ^*ΔΔΔ*^ was also significant (*p* ≤ 0.05, 75%), but at a different magnitude, with a 10% decrease compared to the unamended M_2_. The increased growth, wider aerial mycelia, and number of fruiting bodies at 0.025% H_2_O_2_ in the mutant relative to WT suggest a potentially enhanced sexual reproductive ability under a mild H_2_O_2_ concentration. Nonetheless, this so‐called enhanced sexual reproductive output was overwhelmed by the high concentrations of H_2_O_2_, where dramatically reduced fruiting body numbers in *nph* ^*ΔΔΔ*^ at 0.05% H_2_O_2_ (15%) and 0.1% H_2_O_2_ (10%) were reported, reflecting 95% and 90% significant losses, respectively, in sexual characteristics. It implies that, although the NADPH dehydrogenase mutant can maximize vegetative growth (dense mycelial pigmentation) and sexual reproductive activity by increasing fruiting body production as a way of recognizing and responding to a slight exogenous stressor, elevated exposure levels exacerbate significant oxidative damage in the mutants. Furthermore, the differential effects of H_2_O_2_ concentrations on the mutants, including fruiting body formation enhancement at low levels (0.025%) and severe oxidative damage at higher levels (0.05%–0.1%), justify the dual role of ROS as both signalling molecules (co‐substrate) and cytotoxic agents (oxidative damage‐inducer), as shown in Figure 4C.

**FIGURE 5:**
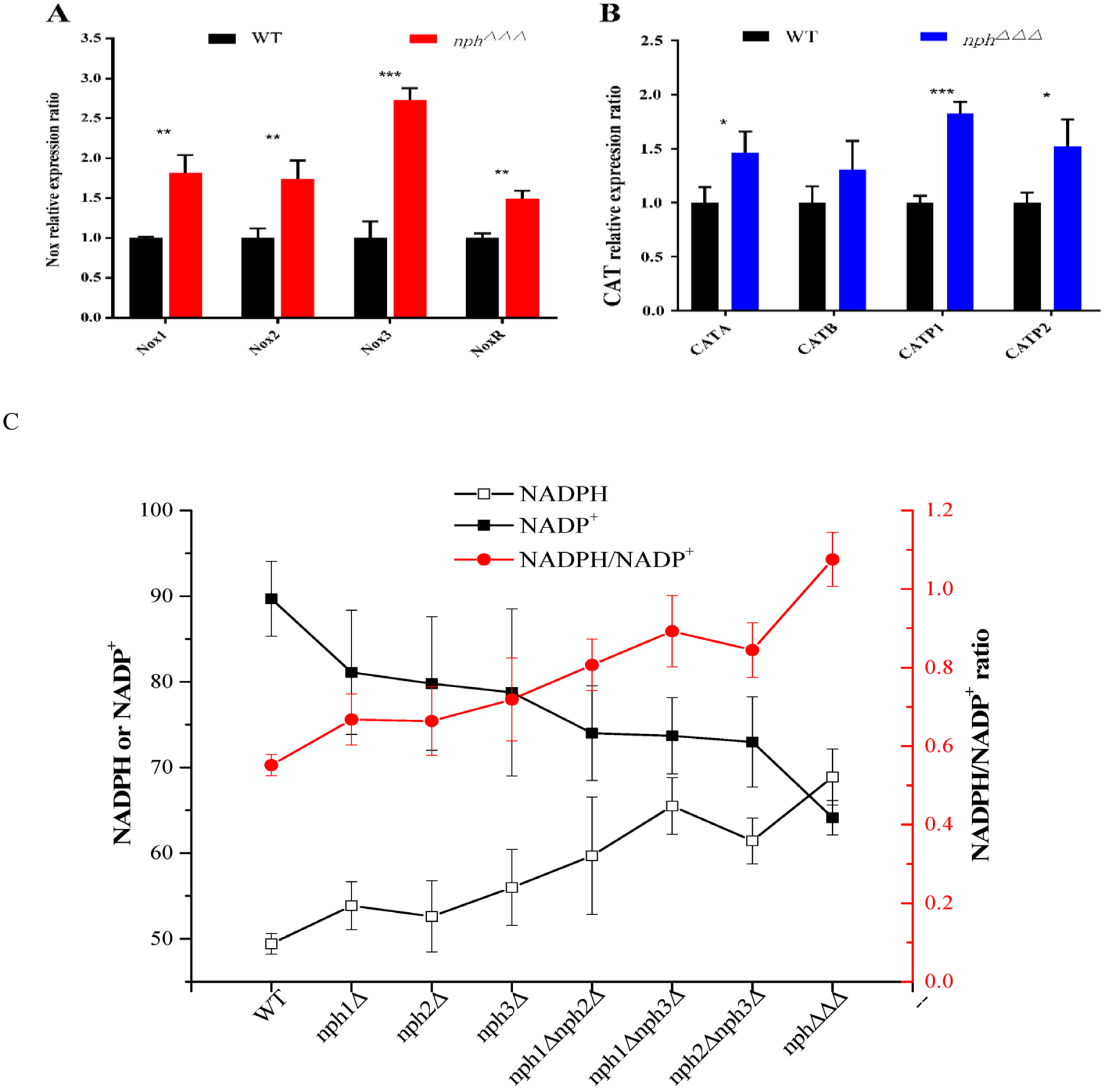
Expression of Nox and CAT genes in wild‐type and triple mutant. The strains were grown in liquid medium for 3 days, and total RNA was extracted. The expression of NADPH oxidase genes (A) and CAT genes (B) was analysed by RT‐qPCR. Results were reported as values for each NADPH oxidase gene normalized to the reference gene PaPDF2, encoding the protein phosphatase PP2A regulatory subunit A, which showed stable expression. (C) Percentage (%) concentration of NADPH, NADP^+^ (μmol/g [dry wt.] mycelia), and the NADPH/NADP^+^ ratios in WT and mutant strains. The mycelia of 2‐day‐old cultures grown on M_2_ medium were harvested and smashed in the extraction buffer. Data are a mean of three replicates, and error bars shown are standard deviations. *t*‐test *p ≤ 0.05, **p ≤ 0.01, ***p ≤ 0.001.

The relative expressions (RE) of Nox and CAT genes were evaluated in the WT and mutant strains. NADPH oxidase (Nox) is an oxidoreductase associated with NADPH oxidation. It is also a major enzyme that produces ROS, which is involved in cell proliferation, differentiation, and defence. At the same time, catalase (CAT) plays a key role in the resistance of cells to ROS. The regulation of NADPH dehydrogenase on ROS was assessed by real‐time quantitative PCR (RT‐qPCR) for Nox and CAT gene expression. For the Nox gene (Figure 5A), four Nox genes were upregulated in the mutant *nph* ^*ΔΔΔ*^ with a significant expression (RE = 2.75) times observed in Nox3, followed by Nox1 (1.80), Nox2 (1.75), and NoxR (1.55), indicating significant up‐regulations in the mutant (*nph* ^*ΔΔΔ*^) relative to WT. The WT showed significantly lower expression of these genes, with a stable relative expression (RE = 1.00) across the different Nox gene homologues. As for the CAT gene (Figure 5B), a similar pattern of up‐regulation was observed with maximum expression (RE = 1.80) in CATP1, followed by CATP2 (1.55), CATA (1.50), and CATB (1.45). As with the Nox, the expression levels of these four CAT genes in the WT (RE = 1.00) remained consistent, indicating functional equivalence among these genes. The expression pattern, which reflected up‐regulation of these genes (Nox and CAT) homologues, could be due to increased oxidative stress triggered by the reduced NADPH availability in the mutant strain as a compensatory response, leading to significantly increased expression of NOX (NADPH oxidase) for ROS production and CAT (catalase) genes for ROS detoxification, initiating a link between ROS signalling and fruiting body formation, given that increased ROS usually stimulates sexual reproductive activity.

### 3.4 Impact of Gene Deletion on Redox Potential, Cellular Respiration, and Fatty Acid Synthesis

The relative intracellular redox potential between WT and mutants, indexed by the NADPH/NADP^+^ ratio, varied considerably. The effect of NADPH dehydrogenase gene deletion on the reducing power of mycelium was ascertained by estimating the NADPH/NADP^+^ ratio of mutants relative to WT (Figure 5C). The concentrations of NADPH and NADP+ in the WT strain were 49.41 ± 1.58 ng/L and 89.69 ± 3.57 ng/L, respectively, suggesting potential depletion/utilization of cellular reducing power. In other words, the significantly increased (*p* ≤ 0.05) NADP^+^ relative to NADPH indicates active cellular metabolism, potentially favouring oxidation over reduction. High concentrations of NADPH were reported in the mutants, while NADP+ levels decreased dramatically, suggesting reduced cellular oxidation and increased reduction processes. Accordingly, the NADPH/NADP^+^ ratio in *nph1* ^*Δ*^, *nph2* ^*Δ*^, and *nph3* ^*Δ*^ mutants increased by 20.6%, 19.7%, and 29.1%, respectively, compared to the WT. Again, the double mutants including *nph1* ^*Δ*^ *nph2* ^*Δ*^, *nph1* ^*Δ*^ *nph3* ^*Δ*^, and *nph2* ^*Δ*^ *nph3* ^*Δ*^ revealed significant increases by 46.4%, 61.3%, and 52.8%, respectively. The triple nph∆∆∆ mutant showed the largest increase, from 0.55 to 1.07, representing a 95.0% increase relative to the wild‐type. Essentially, a significantly increased NADPH: NADP^+^ ratio in the triple nph∆∆∆ mutant, compared to the WT, could be due to loss of NADPH‐consuming enzymatic activity and disruption of ROS‐mediated metabolic flux. The positive Pearson correlation (Figure S2, *r* = 0.961, *p* = 0.0001, and *t* = 8.47) suggests that as the NADPH: NADP^+^ ratio increases, the number of fruiting bodies produced by the *P. anserina* also increases.

**FIGURE 6:**
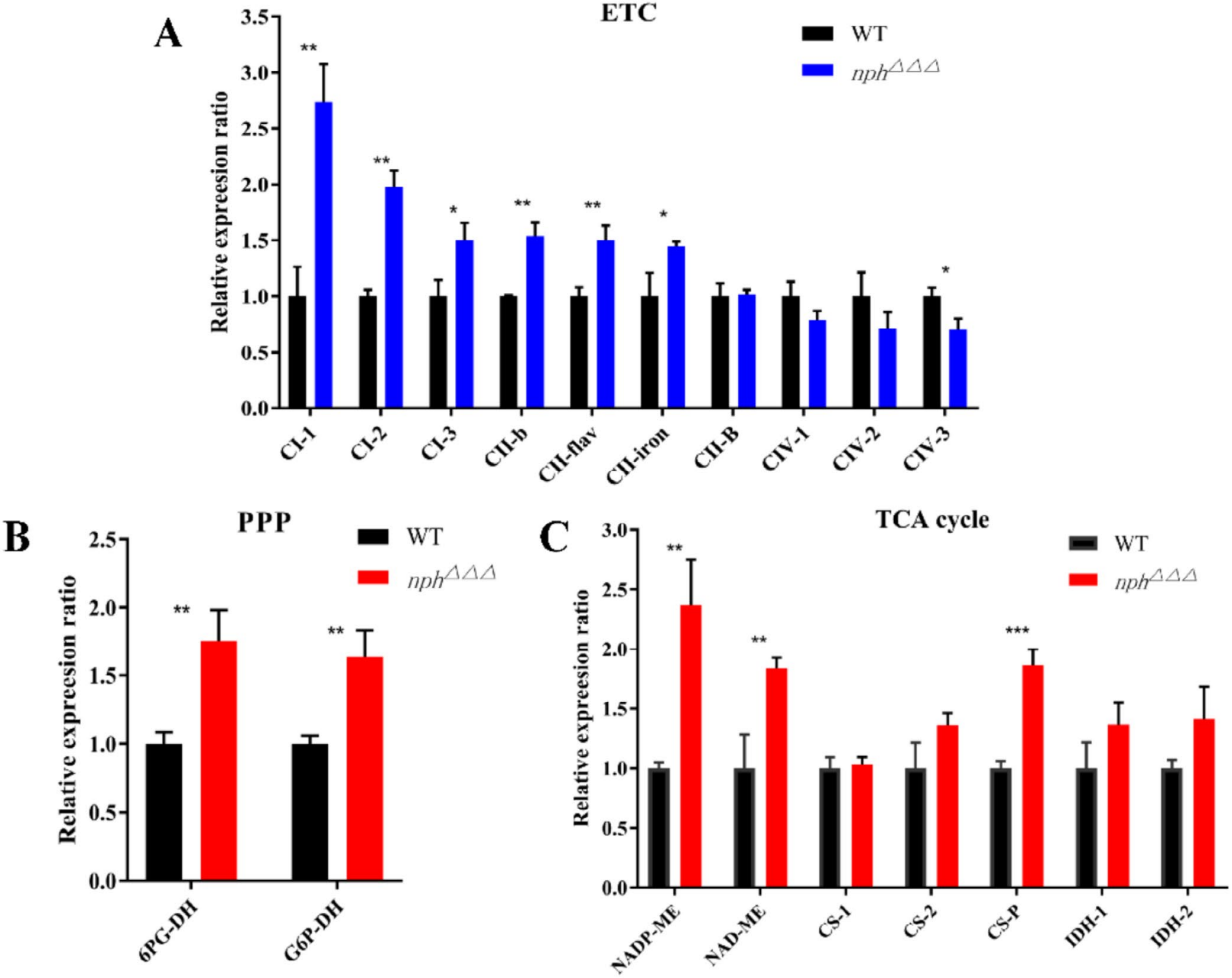
Relative expressions of some important genes in the central metabolic pathway of wild‐type and mutant strains. (A) The expression of some regulatory genes in the electron transport chain (ETC). (B) Expression of key regulatory genes in the pentose phosphate (PPP) pathway. (C) The expression of some regulatory genes in the TCA cycle. Abbreviations: CI, complex I (NADH dehydrogenase); CII, succinate dehydrogenase; CIV, cytochrome c oxidase; G6P‐DH, glucose‐6‐phosphate dehydrogenase; 6PG‐DH, 6‐phosphogluconate dehydrogenase; NADP‐ME, NADP‐dependent malic enzyme; NAD‐ME, NAD‐dependent malic enzyme; CS, citrate synthase; IDH, isocitrate dehydrogenase. Data were normalized by reference gene PaPDF2. Data are a mean of three replicates, and error bars shown are standard deviations. *t*‐test *p ≤ 0.05, **p ≤ 0.01, ***p ≤ 0.001.

Meanwhile, given that redox (NADPH/NADP+) dynamics are linked to central metabolic pathways, including the ETC, PPP, and TCA cycle, as well as antioxidant responses, the potential impact of NADPH dehydrogenase on the expression of the corresponding genes in these pathways in *P. anserina* was evaluated. RT‐qPCR analysis showed significant up‐regulation of some genes in the triple mutant *nph* ^*ΔΔΔ*^. The deletion of NADPH dehydrogenase genes resulted in tremendous enhancement of the transcription level of the NADH dehydrogenase (CI) and succinate dehydrogenase (CII) in the electron transport chain (Figure 6A). This might be a compensatory response to alleviating damage due to the inactivation of NADPH dehydrogenase genes. Nevertheless, the expression of cytochrome c oxidase (CIV) in *nph* ^*ΔΔΔ*^ was significantly decreased, indicating a possible reduction in oxidative phosphorylation (OXPHOS) of the respiratory chain. In contrast, the expression of glucose‐6‐phosphate dehydrogenase (G6P‐DH) and 6‐phosphogluconate dehydrogenase (6PG‐DH) was increased (Figure 6B), suggesting enhanced PPP biosynthetic activity and a strong demand for NADP^+^ as a cofactor. In the TCA cycle, some key enzymes such as malic enzyme (ME) and citrate synthase (CS) were also significantly up‐regulated (Figure 6C). Interestingly, both the PPP pathway and the TCA cycle are sites for substrate‐level phosphorylation (SLP) and NADPH production, indicating that the deletion of NADPH dehydrogenase genes toggled cellular respiration, prioritizing SLP over OXPHOS, allowing SLP to compensate for the corresponding decrease in OXPHOS.

**FIGURE 7:**
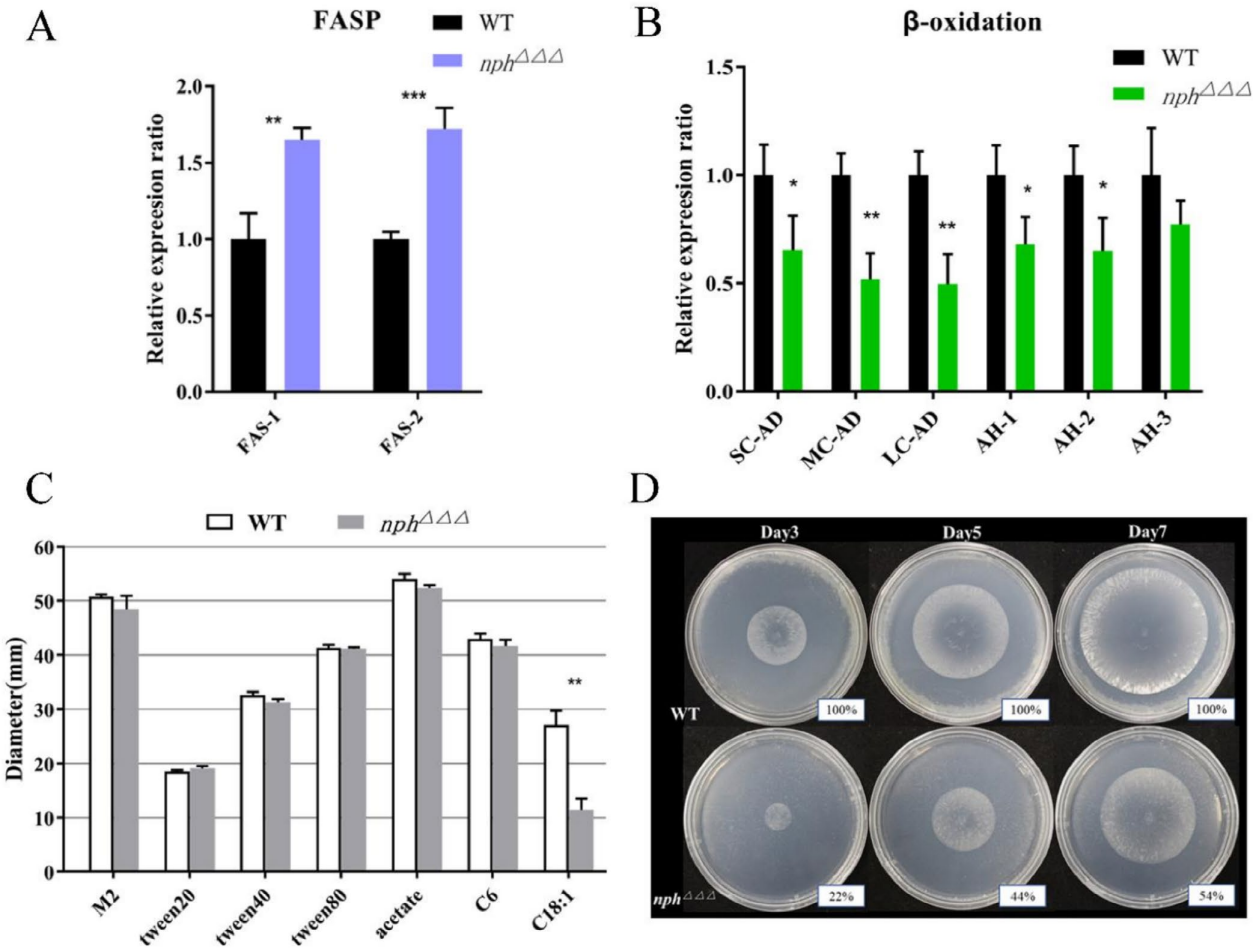
Relative cellular metabolic dynamics in the wild‐type and triple mutant *nph* ^*ΔΔΔ*^ strains. (A) The fatty acid synthase (FAS) expression in fatty acid synthesis pathways (FASP). (B) The expression of important regulatory genes in fatty acid degradation. Abbreviations: SC‐AD, short/branched chain specific acyl‐CoA; MC‐AD, medium‐chain specific acyl‐CoA dehydrogenase; LC‐AD, very long‐chain specific acyl‐CoA dehydrogenase; AH, Enoyl‐CoA hydratase. Expression changes were shown as n‐fold changes in absolute gene regulation relative to the wild‐type strain. Data represent means ± SD of three independent experiments and were normalized by reference gene PaPDF2. Significant changes are indicated by *(*p* < 0.05), ** (*p* < 0.01) and ***(*p* < 0.001). (C) Growing length of wild‐type and *nph* ^*ΔΔΔ*^ on fatty acids. Thalli diameters (mm) for each strain were measured 72 h after incubation on a solid medium at 27°C. The minimal synthetic medium was used and contains either 0.5% (V/V) dextrin (M_2_) or various fatty acids as carbon sources: 1% (V/V) Tween20, 1% (V/V) Tween40, 1% (V/V) Tween80, 50 mM acetate, 20 mM hexanoic acid (C6), and 6 mM oleic acid (C18:1). C18:1 was solubilized with 0.5% (V/V) DMSO. When required, the pH was adjusted to 7. Data are a mean of three replicates, and error bars shown are standard deviations. *t*‐test *p ≤ 0.05, **p ≤ 0.01, ***p ≤ 0.001. (D) Colony morphology of wild‐type and triple mutant *nph* ^*ΔΔΔ*^ on oleic acid medium. Images of mycelia from mutant and wild‐type strains were taken after 3, 5, and 7 days of incubation. Growth of the triple mutant was reduced on oleic acid medium, and the reduction was expressed as a percentage relative to WT.

**FIGURE 8:**
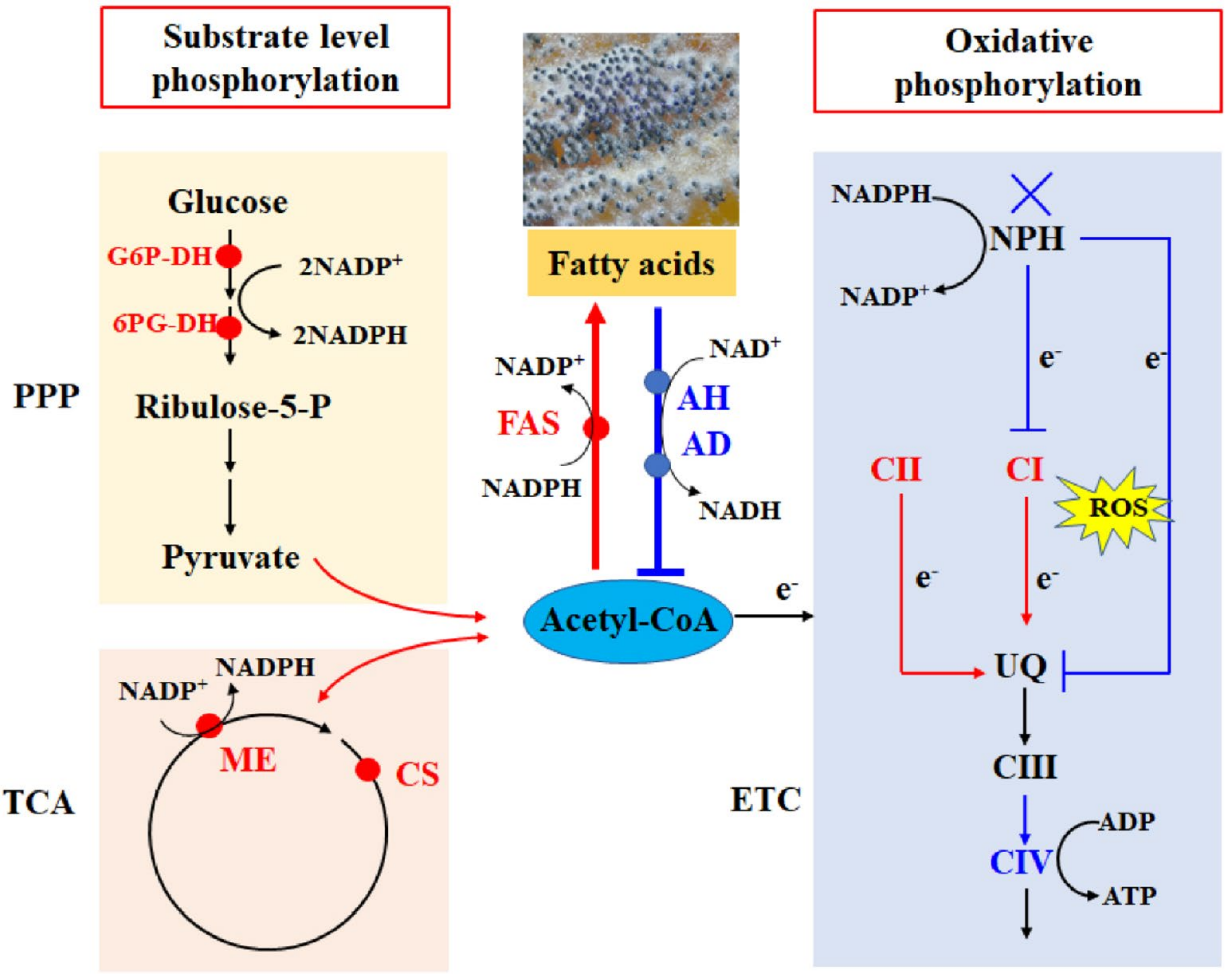
Illustrative model for interpreting the rearrangement of energy metabolism due to the NADPH dehydrogenase deficiency. Deletion of NADPH dehydrogenase genes reduces intracellular oxidative phosphorylation, and cells maintain energy balance by increasing substrate‐level phosphorylation. Meanwhile, intracellular reducing power increases, promoting fatty acid synthesis and decreasing catabolism. 6PG‐DH, 6‐phosphogluconate dehydrogenase; CI, NADH dehydrogenase; CII, succinate dehydrogenase; CIII, ubiquinol‐cytochrome bc1 reductase; CIV, cytochrome c oxidase; CS, citrate synthase; FAS, fatty acid synthase; G6P‐DH, glucose‐6‐phosphate dehydrogenase; ME, malic enzyme; NPH, NADPH dehydrogenase; PPP, pentose phosphate pathway; Ribulose‐5‐P, Ribulose‐5‐phosphate; TCA, tricarboxylic acid cycle; UQ, CoQ/ubiquinone pool.

Additionally, energy consumption characteristics, as indexed by gene expression associated with fatty acid metabolism, were used to assess cellular metabolic rates during fruiting body formation. Our results revealed that the expression level of fatty acid synthase (FAS) in the mutant strain was significantly increased (*p* ≤ 0.001, Figure 7A). However, genes related to *β*‐oxidation, such as acyl‐CoA dehydrogenase (AD) and Enoyl‐CoA hydratase (AH), were down‐regulated (Figure 7B). Moreover, the growth of the triple mutant was inhibited by oleic acid as the sole carbon source, and the growth diameter was significantly reduced relative to WT (Figure 7C). On the third day, the colony diameter observed in the *nph* ^*ΔΔΔ*^ mutant was 22% of WT. On the fifth and seventh days, the colony diameters of the mutant *nph* ^*ΔΔΔ*^ were reduced by 56% and 46%, respectively (Figure 7D). The mechanistic model linking energy distribution processes, SLP, OXPHOS, fatty acid metabolism, and the central metabolic pathways is illustrated in Figure 8.

## 4 Discussion

Several NADPH dehydrogenases in plants and filamentous Ascomycetes have been purified and characterized during the last decades (Melo et al. 2001; Michalecka et al. 2004). To the best of our knowledge, there have been scarce reports on the involvement of NADPH dehydrogenase during fruiting body development in *P. anserina*. Until this study, the mechanisms regulating energy distribution and fatty acid metabolism by NADPH dehydrogenase have received little investigation. The present study hence evaluated the role of NADPH dehydrogenase in fruiting body formation by regulating energy distribution and fatty acid metabolism. As a result, three NADPH dehydrogenase sequences from the genome database were obtained, which were highly similar to NADPH dehydrogenases in fungi, *O. piceae*, and *C. cinerea*, as presented in Figure 1.

The relative enzyme activity varied considerably among the mutant strains. Deletion of NADPH dehydrogenase resulted in a significant decrease in enzyme activity. This was especially true for the double and triple mutants (*p* ≤ 0.001); however, fruiting body numbers were significantly increased, suggesting complex metabolic trade‐offs. Specifically, it is recalled that NADPH is a key cofactor involved in a wide range of cellular metabolisms. First, biosynthetic reactions, including nucleotide, amino acid, and lipid synthesis. Second, redox homeostasis and mitochondrial function; third, antioxidant defence, which protects biological systems against oxidative stress. In other words, gene inactivation affecting NADPH metabolism would usually disrupt any enzymatic reaction that requires NADPH dehydrogenase (Maas et al. 2010). Hence, the dramatically reduced enzyme activity reported in the mutants is largely linked to the low availability of NADPH‐dependent cofactors. The effect of the gene deletion on fruiting body numbers showed a linear relationship, with more fruiting bodies reported as the number of mutant strains increased. A possible reason could be linked to increased oxidative and/or metabolic stress, which may initiate stress‐induced reproductive responses in the mutants. Again, the NADPH gene deletion may have altered intracellular energy allocation, greatly favouring reproductive over vegetative growth, thereby promoting greater fruiting body development in the mutant in response to mitochondrial signalling modifications. Essentially, this phenomenon is a classic life‐history trade‐off, in which fungal species under stress quickly prioritize sexual reproduction (fruiting body formation) over maintenance (vegetative growth) to maximize survival under challenging conditions (Pontzer and McGrosky 2022). Similarly, a previous study reported that the NADPH Oxidase Gene, *FgNoxD*, inactivation in *Fusarium graminearum* reduced vegetative growth and conidia production, although, unlike our study, sexual development was restrained in the mutant (Li et al. 2022), indicating slight variabilities in metabolic and genetic responses to external triggers among different filamentous fungi. Thus, ROS plays an important role in cellular energy metabolism and regulatory networks (Lennicke and Cochemé 2021). As it is well known that ROS are mainly produced by the respiratory chain, which largely influences fruiting body formation, we hypothesized that deleting NADPH dehydrogenase genes would affect fruiting body development by altering ROS regulation in *P. anserina* . The response of mutants to increasing H_2_O_2_ concentrations in the present study showed that elevated ROS levels can significantly increase susceptibility to oxidative stress, leading to severe oxidative damage. Additionally, it has been demonstrated that ROS derived from Nox2 can mediate cell dysregulation and aging, leading to oxidative stress (Schieber and Chandel 2014). However, a mild concentration of H_2_O_2_ is needed by *P. anserina* to maximize both oxidative tolerance and fruiting body formation, as shown in Figure 4, where a 0.025% H_2_O_2_ led to more fruiting body numbers in the mutant over WT. In other words, H_2_O_2_ can act as a co‐substrate at low concentration and pose a potential threat to cellular stability at high concentration. Furthermore, it has been reported that mild oxidative stress could trigger mitohormesis, a mitochondrial stress‐induced increment in health and cellular viability, as a protective stress response that potentially promotes reproduction and encourages lifespan extension (Knuppertz et al. 2017). The homologues of the antioxidant genes (Nox and CAT) exhibited uniform down‐regulation in the WT, as presented in Figure 5A,B, confirming NADPH availability and thus limited triggers for the regulation of the genes; however, in the NADPH‐deficient mutant, these genes were significantly up‐regulated as a direct response to nullify the stress due to the NADPH gene inactivation. This demonstrates that Nox and CAT are regulatory genes and are thus produced in response to cellular need. The absence of the NADPH gene in the mutant led to increased expression of antioxidant genes, conferring emergency resistance to the induced stress. The expression of Nox1 is consistent with the increasing trend of fruiting body formation in the mutant. Indeed, Nox1 is closely associated with fungal sexual development by enhancing resource mobilization from neighbouring cells (Takemoto et al. 2007). Sexual reproduction eventually becomes a protective mechanism against reactive oxygen species and resultant DNA damage (Wallen and Perlin 2018). The NADPH dehydrogenase is an important regulator of energy homeostasis and is highly conserved across eukaryotic organisms (Chen et al. 2024). While Nox, a multimeric enzyme and flavoprotein, generates ROS as a mediator of physiological mechanisms (Tudzynski et al. 2012), the CAT, a tetrameric enzyme consisting of 4 polypeptides with porphyrin heme‐iron groups, acts accordingly to decompose the H_2_O_2_ into H_2_O and O_2_ for protection against cellular damage (Kirkman and Gaetani 1984). In essence, the significant (*p* ≤ 0.001) expressions of Nox and CAT genes in the NADPH dehydrogenase mutant are associated with oxidative stress caused by reduced NADPH availability, allowing the cell to compensate by increasing ROS production (via Nox) to activate stress signalling and increasing ROS detoxification (via CAT), thereby effectively preventing potential oxidative damage. Previous studies have characterized the related oxidoreductases and identified the role of NADPH oxidases in ROS and cellular signalling in other filamentous fungi, including *Fusarium* (Fernando et al. 2018; Nordzieke et al. 2019), *Aspergillus* and *Cryptococcus species* (Hogan and Wheeler 2014). Again, the NADPH oxidase‐generated ROS have been shown to regulate sexual development in *Aspergillus nidulans*, confirming the critical roles of NADPH in fungal physiology and differentiation (Lara‐Ortiz et al. 2003).

The NADPH/NADP+ ratio is used as an index of the extent of NADPH oxidation to NADP+ via NADPH dehydrogenase. The intracellular redox potential is determined by the NADPH/NADP^+^ ratio (Zhang et al. 2015). In the present study, the NADPH/NADP+ ratio in the triple mutant increased significantly, indicating a greater reductive than oxidative burden, which warrants significant up‐regulation of antioxidant genes. Notwithstanding, the NADPH controls biosynthetic pathways activation by fueling anabolic reactions, including nucleotide (like DNA and RNA replications), amino acid metabolism (especially for growth and reproduction), and fatty acid biosynthesis (e.g., membrane and lipid storage); hence, an NADPH > NADP^+^ redox status would potentially enhance fungal growth due to increased biosynthesis (Lee et al. 2010). The high Pearson correlation coefficient, as shown in Figure S2 indicated an extremely positive correlation between fruiting body formation and NADPH: NADP dynamics. Nonetheless, the elevated NADPH: NADP+ ratio reported in the mutant may not solely reflect excessive NADPH production but also accumulation. This is because NADPH: NADP^+^ is closely controlled by metabolic feedback loops. Specifically, when the NADPH‐consuming pathway is affected, e.g., due to gene inactivation as in our study, the cell experiences NADPH accumulation (Li et al. 2021) without corresponding NADP^+^ processing, thereby initiating metabolic feedback disruptions and preventing proper NADPH utilization (Li et al. 2021).

Responses to environmental uncertainties and intracellular abnormal metabolisms usually prompt organisms to allocate resources to growth or reproductive processes (Finstad et al. 2010). NADPH dehydrogenase is an enzyme involved in the respiratory chain, and its reaction contributes to energy conservation (Xiao et al. 2018). The present study demonstrated that one of the survival strategies for NADPH mutants is to transfer more substances and energy to the fruiting body formation by increasing intracellular reducing capacity. Intrinsically, the NADPH dehydrogenase mutants inhibited the electron transport chain (ETC), leading to decreased OXPHOS coupling. Regardless, potential energy reallocation involving SLP, particularly the PPP pathway and the TCA cycle, was increased in *nphΔΔΔ* strains, thereby enhancing fatty acid biosynthesis and promoting fruiting body formation. Similarly, up‐regulation of many genes associated with fatty acid metabolism during fruiting body formation has been previously reported, demonstrating that most fungi exhibit “smart” regulatory mechanisms to counter metabolic abnormalities in cells. It accentuates the significant role of NADPH dehydrogenase during fruiting body formation in *P. anserina* . Again, the production of more fruiting bodies in the NADPH mutant strain can be attributed to NADPH acting as a cofactor to initiate fatty acid biosynthesis. Therefore, a higher cytosolic NADPH/NADP^+^ ratio (i.e., high reducing power) benefits the biosynthesis of fatty acids (Fatihi et al. 2015). Furthermore, the PPP pathway is a major source of NADPH, providing ribulose‐5‐phosphate for fatty acid biosynthesis (Lane and Fan 2015). Fruiting body formation is closely linked to cellular biosynthesis, especially fatty acids (He et al. 2023). Most targeted gene knockouts impact lipid accumulation considerably. Specifically, while the knockout of *CAR1* in the oleaginous filamentous fungus *Mucor circinelloides* reduced lipid biosynthesis (Ramadan et al. 2026), the deletion of *Snfα1* in the same dimorphic fungus led to a 27.20% increase in lipid accumulation (Pang et al. 2025). This is consistent with our results, where an increase in fruiting bodies in the NADPH mutant is accompanied by the up‐regulation of genes associated with fatty acid synthesis, as shown in Figure 7A. The main form of fatty acid degradation is *β*‐oxidation, which mainly occurs in the peroxisomes and mitochondria (Poirier et al. 2006). Mitochondria and peroxisomes in cells can be targeted by a long C‐terminal sequence in NADPH dehydrogenase (Wallström et al. 2014). The mitochondrial *β*‐oxidation pathway in mutant strains (*ΔechA*) showed a deficient phenotype on a medium containing oleic acid as the sole carbon source (Boisnard et al. 2009). Our results showed that NADPH mutant strains also exhibited reduced growth on medium containing oleic acid as the sole carbon source, consistent with the down‐regulation of *β*‐oxidative genes. Largely, NADPH dehydrogenase mutation enhanced fatty acid biosynthesis while inhibiting the pathway of fatty acid degradation, so that the mycelia of the NADPH mutants could utilize more substances and energy to produce fruiting bodies proportionately. It has been reported that energy metabolism is reprogrammed in cells in response to endogenous or exogenous stimuli, thereby disrupting the original metabolic balance and altering intracellular reducing power (Pestoni et al. 2019). The reducing power, such as NADPH/NADP^+^, can determine the intracellular redox potential, which affects the thermodynamic driving force of many reactions in vivo (Liu et al. 2013). Both OXPHOS and SLP are cellular respiration processes that maintain and regulate reducing power (Wilson 2017). The electron flux capacity of the ETC is governed by CIV activity, otherwise known as cytochrome c, and it can use molecular oxygen (O_2_) as the terminal electron acceptor. This primarily controls the efficiency of coupling mitochondrial OXPHOS (Oliva et al. 2011). Reduced CIV activity in NADPH dehydrogenase mutants indicated decreased ETC electron flux, leading to reduced OXPHOS coupling. Electrons from CI, CII, and NADPH dehydrogenase can be accepted by Coenzyme Q (ubiquinone; UQ) (Gnaiger 2024). The present study shows that both CI and CII levels were elevated in NADPH dehydrogenase gene‐deficient strains, suggesting that these strains may trigger compensatory pathways to maintain electron flux. Accordingly, the complex activities of ETC could be remodelled in the cells to improve the bioenergetic reserve capacity. The reduced OXPHOS level in NADPH dehydrogenase mutants disrupted intracellular metabolic balance. Moreover, the remodelling of ETC is closely related to cellular metabolic transformation (Schwimmer et al. 2005). Consequently, the SLP is also another energy metabolic pathway involved in cellular metabolic networks of eukaryotic non‐photosynthetic organisms (Kiss et al. 2014). Our results showed that the PPP pathway and the TCA cycle were enhanced in the mutants, suggesting that the main response mechanism of the NADPH mutant to ETC remodelling is an increase in SLP over OXPHOS. Interestingly, SLP not only provides energy to support cellular energy metabolism but also increases cellular reducing power (Hunt et al. 2010). The results indicated that the ratio of intracellular NADPH/NADP^+^ in the mutants, especially in *nph* ^*ΔΔΔ*^ was increased by more than double. Similarly, suppression of the *StNDB1* gene has been shown to increase the NADPH/NADP^+^ ratio in *N. sylvestris* (Liu et al. 2008). In effect, the reprogramming of energy metabolism in cells was caused by the deletion of the NADPH dehydrogenase gene. The intracellular reducing capacity can be improved primarily by regulating the balance between OXPHOS and SLP. Previous studies reported that low CIV activity potentially made cells more susceptible to the injury and oxidative stress (Oliva et al. 2017). Besides, it has been shown that the enhancement in intracellular ROS production was caused by suppression of CIV expression (Li Campian et al. 2007). The increase in the amount of CI, CII, and the decrease in CIV in the respiratory chain indicated potential remodelling of the intracellular ROS signalling.

Increased SLP over OXPHOS due to NADPH deletion intensified the disruptions in mitochondrial redox balance and ATP production, as illustrated in Figure 8. NADPH dehydrogenases usually transfer electrons from NADPH to the mitochondrial electron transport chain, thereby driving ATP generation via OXPHOS. However, once the gene encoding NADPH is deleted, its ability to donate electrons to the ETC would be significantly reduced (Malagnac et al. 2004). Hence, contributing less proton pumping across the mitochondrial membrane in addition to other cellular dynamics, including near‐total reliance on alternative ATP‐generating mechanisms, like SLP (glycolysis, PPP, and TCA cycle), reduced ATP production via OXPHOS, minimal O_2_ consumption and electron flow through ETC, and enhancing fatty acid biosynthesis by up‐regulations of related genes. These phenomena are beneficial to *P. anserina* because they help ensure survival under mitochondrial dysfunction and in stressed conditions, as SLP supports rapid ATP production, operates in anaerobic conditions, is independent of the proton gradient and membrane potential, and limits oxidative stress by reducing ROS production. Although SLP produces less ATP per glucose molecule than OXPHOS (Rigoulet et al. 2020), the numerous advantages elaborated above make SLP a crucial survival strategy employed by *P. anserina* to maximize fruiting body formation in stressed conditions.

## 5 Conclusion

In this study, we demonstrated that NADPH dehydrogenase plays a central regulatory role in fruiting body formation, energy homeostasis, redox balance, and fatty acid metabolism in *P. anserina* . Deletion of NADPH dehydrogenase genes significantly reduced enzyme activity, particularly in double and triple mutants, yet paradoxically increased fruiting body formation, suggesting compensatory metabolic reprogramming that prioritises reproductive development over vegetative maintenance. The mutants showed increased sensitivity to elevated H_2_O_2_ concentrations, confirming disruption of intracellular redox homeostasis; however, mild oxidative stress stimulated fruiting body formation. Up‐regulation of antioxidant genes, particularly Nox and CAT homologues, further demonstrated activation of ROS‐mediated stress signalling pathways in response to NADPH dehydrogenase deficiency. Furthermore, the significantly elevated NADPH/NADP^+^ ratio in the mutants reflected reduced reducing power and impaired NADPH utilisation resulting from the disruption of NADPH‐consuming pathways.

Metabolic analyses revealed extensive remodelling of cellular energy metabolism following NADPH dehydrogenase gene deletion. Reduced cytochrome c oxidase activity indicated suppression of oxidative phosphorylation (OXPHOS), whereas increased activities of Complex I and II, together with enhanced fluxes through the pentose phosphate pathway and the tricarboxylic acid cycle, suggested compensatory enhancement of substrate‐level phosphorylation (SLP). This metabolic shift increased intracellular reducing capacity and promoted fatty acid biosynthesis while suppressing *β*‐oxidation. Consequently, more cellular resources and energy were redirected toward reproductive development, as evidenced by up‐regulation of fatty acid biosynthetic genes and reduced utilization of oleic acid as the sole carbon source. The present study provides new mechanistic insights into how metabolic reprogramming enables *P. anserina* to maximize fruiting body formation under mitochondrial and oxidative stress conditions.

## Author Contributions

**Yanling Qiu:** conceptualization, investigation, writing – original draft, methodology, visualization, software, formal analysis, data curation. **Amechi S. Nwankwegu:** writing – original draft, methodology, visualization, formal analysis. **Weizhao Chen:** visualization, formal analysis. **Yuanjing Li:** visualization, formal analysis, methodology. **Gang Liu:** supervision, visualization. **Yue Sun:** formal analysis, methodology, visualization. **Ning Xie:** conceptualization, funding acquisition, writing – review and editing, supervision.

## Funding

This work was supported by Basic and Applied Basic Research Foundation of Guangdong Province, 2024B1515020034. Shenzhen Science and Technology Program, JCYJ20250604182001002. National Key Research and Development Program of China, 2021YFA0910800. Basic and Applied Basic Research Fund of Guangdong Province, 2021A1515012166, 2021A1515012118. National Natural Science Foundation of China, 31601014, 22078199.

## Conflicts of Interest

The authors declare no conflicts of interest.

## Supporting information


**Figure S1:** Southern blotting analysis of the ^*Δ*^ *nph* mutants. DNA was isolated from wild‐type *P. anserina* and from one or more purified transformants, and digested with appropriate restriction enzymes. The blots were probed with a sequence containing the relevant coding sequence (CDS) and its flanking regions. For each NADPH dehydrogenase gene, a restriction map of the wild‐type and mutant locus is presented. The sizes of the expected fragments are indicated on the maps and are reported close to the corresponding fragment on the Southern blot.
**Figure S2:** Linear correlation clarifying the mechanistic interactions between the number of fruiting bodies and the fluctuations in NADPH: NADP^+^ ratios between WT and mutant strains. Pearson correlation (*r*) = 0.961, N (i.e., WT, *nph1* ^*Δ*^, *nph2* ^*Δ*^, *nph3* ^*Δ*^, *nph1* ^*Δ*^ *nph2* ^*Δ*^, *nph1* ^*Δ*^ *nph3* ^*Δ*^, *nph2* ^*Δ*^ *nph3* ^*Δ*^, and *nph* ^*ΔΔΔ*^) = 8, DF = 6, *p*‐value = 0.0001, and *t*‐statistic = 8.47, where “*t*” is mathematically expressed as: $t=r\;x\;\frac{\sqrt{n-2}}{\sqrt{1-r^{2}}}$, DF = Degree of freedom. The Pearson correlation coefficient *r* = 0.961 (*r* ^2^ ≈0.923) between the number of fruiting bodies and the NADPH: NADP^+^ ratio indicates a very strong positive linear relationship, and the biological implication demonstrates that higher intracellular reducing power (i.e., higher NADPH relative to NADP^+^) is strongly associated with increased fruiting body formation in *P. anserina* . In other words, assuming a linear model, such a high *r* ^2^ value implies that 92.3% of the variation in fruiting body number in *P. anserina* can be driven by NADPH: NADP^+^ dynamics.
**Table S1:** NADPH dehydrogenase genes of *P. anserina.*

**Table S2:** Primers for gene deletion and detection using the split‐marker approach.
**Table S3:** Primers for Real‐Time RT Quantitative. PCR. PDF2 (protein phosphatase PP2A regulatory subunit A, Pa_7_6690) was used as a normalization reference gene. Each real‐time amplification reaction contained 0.5 ng of cDNA, 0.5 μM of primers, and 10 μL of 2 × SYBR TB Green enzyme mix, for a final volume of 20 μL. The relative quantitative analysis was performed with the 2^−△△Ct^ method, in which ^△^Ct = Ct target gene − Ct internal reference gene, ^△△^Ct = ^△^Ct sample−△Ct control.

## Data Availability

Data are available on request from the authors.
