# Supplementary material for: NADPH Dehydrogenase Gene Regulates Energy Distribution and Fatty Acid Metabolism During Fruiting Body Formation in the Filamentous Fungus Podospora anserina
Source: Microb Biotechnol. 2026 Jun 15;19(6):e70401. doi: 10.1111/1751-7915.70401 (PMC13269838; doi:10.1111/1751-7915.70401)
Supplement: Supplementary file 1 — Figure S1: Southern blotting analysis of the Δ nph mutants. DNA was isolated from wild‐type P. anserina and from one or more purified transformants, and digested with appropriate restriction enzymes. The blots were probed with a sequence containing the relevant coding sequence (CDS) and its flanking regions. For each NADPH dehydrogenase gene, a restriction map of the wild‐type and mutant locus is presented. The sizes of the expected fragments are indicated on the maps and are reported close to the corresponding fragment on the Southern blot. Figure S2: Linear correlation clarifying the mechanistic interactions between the number of fruiting bodies and the fluctuations in NADPH: NADP+ ratios between WT and mutant strains. Pearson correlation (r) = 0.961, N (i.e., WT, nph1 Δ , nph2 Δ , nph3 Δ , nph1 Δ nph2 Δ , nph1 Δ nph3 Δ , nph2 Δ nph3 Δ , and nph ΔΔΔ ) = 8, DF = 6, p‐value = 0.0001, and t‐statistic = 8.47, where “t” is mathematically expressed as: t=rxn−21−r2, DF = Degree of freedom. The Pearson correlation coefficient r = 0.961 (r 2 ≈0.923) between the number of fruiting bodies and the NADPH: NADP+ ratio indicates a very strong positive linear relationship, and the biological implication demonstrates that higher intracellular reducing power (i.e., higher NADPH relative to NADP+) is strongly associated with increased fruiting body formation in P. anserina . In other words, assuming a linear model, such a high r 2 value implies that 92.3% of the variation in fruiting body number in P. anserina can be driven by NADPH: NADP+ dynamics. Table S1: NADPH dehydrogenase genes of P. anserina. Table S2: Primers for gene deletion and detection using the split‐marker approach. Table S3: Primers for Real‐Time RT Quantitative. PCR. PDF2 (protein phosphatase PP2A regulatory subunit A, Pa_7_6690) was used as a normalization reference gene. Each real‐time amplification reaction contained 0.5 ng of cDNA, 0.5 μM of primers, and 10 μL of 2 × SYBR TB Green enzyme mix, for a final volume [file MBT2-19-e70401-s001.docx]

**Table S1.**

NADPH dehydrogenase genes of *P. anserina*

| Gene | Locus | Accession ^a^ | No. Protein Length^b^ |
| --- | --- | --- | --- |
| nph1 | Pa_1_9760 | CAP69312 | 426 |
| nph2 | Pa_4_60 | CAP61362 | 416 |
| nph3 | Pa_6_6330 | CAP71674 | 383 |

^a^ cDNA sequence. ^b^ Length of proteins is given in amino acid residues without considering putative posttranslational processing.

**Table S2.**

Primers for gene deletion and detection using the split-marker approach.

| Primer name | Sequence (5'->3') | Description |
| --- | --- | --- |
| Panph1-1F | CTCGCGTCATGTTTCGAGTT | deletion of *nph1*(Pa_1_9760) |
| Panph1-2R | CTATTTAACGACCCTGCCCTGAACCGAACAAGGTAAAGCCGGCAGT | deletion of *nph1*(Pa_1_9760) |
| Panph1-mkF | ACTGCCGGCTTTACCTTGTTcggttcagggcagggtcgttaaatag | deletion of *nph1*(Pa_1_9760) |
| Panph1-mkR | CTTGGGAACAAGAACGGGCTCATCGAACTGGATCTCAACAGCGGTAAG | deletion of *nph1*(Pa_1_9760) |
| Panph1-3F | CTTACCGCTGTTGAGATCCAGTTCGATGagcccgttcttgttcccaag | deletion of *nph1*(Pa_1_9760) |
| Panph1-4R | ATGAGCACGATTGGGTGGAG | deletion of *nph1*(Pa_1_9760) |
| Panph1-5Test | agcgaacacccgttcaaggt | detection of the deletion of *nph1* |
| Panph1-3Test | CAGAAAGGGTCAGGCTACCAAG | detection of the deletion of *nph1* |
| Panph2-1F | AATGTACCTGGCCATCGGTG | deletion of *nph2* (Pa_4_60) |
| Panph2-2R | CTATTTAACGACCCTGCCCTGAACCGCTTCAGACCGAAACTCGTCG | deletion of *nph2* (Pa_4_60) |
| Panph2-mkF | CGACGAGTTTCGGTCTGAAGcggttcagggcagggtcgttaaatag | deletion of *nph2* (Pa_4_60) |
| Panph2-mkR | ACACAGCAAAGGGAGCTCAGcatcgaactggatctcaacagcggtaag | deletion of *nph2* (Pa_4_60) |
| Panph2-3F | CTTACCGCTGTTGAGATCCAGTTCGATGCTGAGCTCCCTTTGCTGTGT | deletion of *nph2* (Pa_4_60) |
| Panph2-4R | ACCTTGCTGATCCACAGGCT | deletion of *nph2* (Pa_4_60) |
| Panph2-5Test | AGATGATGGAAGGCCTCGGT | Detection of the deletion of *nph2* |
| Panph2-3Test | CAGTCTCTGAGCGAGTGGCT | detection of the deletion of *nph2* |
| Panph3-1F | AAGTCGTCGTTGGTTGGGAT | deletion of *nph3* (Pa_6_6330) |
| Panph3-2R | CTATTTAACGACCCTGCCCTGAACCGAGTTGGGTGAGGGTGAAGTG | deletion of *nph3* (Pa_6_6330) |
| Panph3-mkF | CACTTCACCCTCACCCAACTcggttcagggcagggtcgttaaatag | deletion of *nph3* (Pa_6_6330) |
| Panph3-mkR | CTTTATCCAACCCGCTCGCTcatcgaactggatctcaacagcggtaag | deletion of *nph3* (Pa_6_6330) |
| Panph3-3F | CTTACCGCTGTTGAGATCCAGTTCGATGAGCGAGCGGGTTGGATAAAG | deletion of *nph3* (Pa_6_6330) |
| Panph3-4R | ACAATTGGTTCGGTGCAGCT | deletion of *nph3* (Pa_6_6330) |
| Panph3-5Test | cgactggaactttggacgat | Detection of the deletion of *nph3* |
| Panph3-3Test | CGTGGTTTCGCAGATCTGTT | Detection of the deletion of *nph3* |
| Common 5Test | TGAGAAGCACACGGTCAC | detection of the deletion mutant |
| Common 3Test | TCGGGGCGAAAACTCTC | detection of the deletion mutant |

**Table S3.**

Primers for Real-Time RT Quantitative. PCR. PDF2 (protein phosphatase PP2A regulatory subunit A, Pa_7_6690) was used as a normalization reference gene. Each real-time amplification reaction contained 0.5 ng of cDNA, 0.5 µM of primers, and 10 µL of 2 × SYBR TB Green enzyme mix, for a final volume of 20 µL. The relative quantitative analysis was performed with the 2^−△△Ct^ method, in which ^△^Ct = Ct target gene − Ct internal reference gene, ^△△^Ct = ^△^Ct sample − ^△^Ct control.

| **Primer name** | **Sequence (5'->3')** | **Description** |
| --- | --- | --- |
| PaPDF2-F | GCAGACAGGTTCGAAAAGATTG | Exon4-5 |
| PaPDF2-R | CAGATGATCAATGGTTTCTTGC | Exon5-6 |
| PaNox1_F | GGGAGCACTGCATTGGTTAT | Nox1 |
| PaNox1_R | CGACGTCATACGGATGTTTG | Nox1 |
| PaNox3_F | AACGGTCAAGAGCTGGAAGA | Nox3 |
| PaNox3_R | GCCACTACCAGCCCAATAAA | Nox3 |
| PaNox2_F | TCACCCATTCACTCTGACCA | Nox2 |
| PaNox2_R | ACACCAACAACCTTGGAAGC | Nox2 |
| PaNoxR_F | CAACGACGACCTCTACGACA | NoxR |
| PaNoxR_R | GTCGTAGTCGCTGCCTTCTTC | NoxR |
| PaCATA_F | GCGGTGTTGATTCCATTCGC | CATA |
| PaCATA_R | ACTAACAGCTGGCAGGCCG | CATA |
| PaCATB_F | CTACCATAACAATGTCACGGCTG | CATB |
| PaCATB_R | CCTTGCTGAGCTGCTCCTTC | CATB |
| PaCATP1_F | TTCCGCTCTCTTCGTCTCGG | CATP1 |
| PaCATP1_R | CATTCTTGGCAAAGACCTTGTCT | CATP1 |
| PaCATP2_F | GTATGCGCCCAACAGCTTTG | CATP2 |
| PaCATP2_R | AGCTGTGTCGCCTGCGCA | CATP2 |
| PaCI-1_F | GTACACGATCCAGCTGGAGAACG | CI-1 |
| PaCI-1_R | CCAGTCACATGAAGGAACTCATCC | CI-1 |
| PaCI-2_F | GCTGTCGTCAGGACACATACTACC | CI-2 |
| PaCI-2_R | CACCTCCCTTGGCCTCTCTG | CI-2 |
| PaCI-3_F | TGCCAAGCACGACTACGATG | CI-3 |
| PaCI-3_R | TGTGCTCGGTGTTGGCCC | CI-3 |
| PaCII-b_F | CACCCTCCTCATCCACTCTC | CII-b |
| PaCII-b_R | ATCGCTGGTCTCGTACTCGT | CII-b |
| PaCII-iron_F | AAGGATCTCGTTCCCGATCT | CII-iron |
| PaCII-iron_R | CGTAAAGACCGTCGAGCTTC | CII-iron |
| PaCII-flav_F | CCTTGTTGAGACCCTTGAGC | CII-flav |
| PaCII-flav_R | TGTGCTTCATCCAGTTCTCG | CII-flav |
| PaCII-B_F | GGCGGCCTCTACGTCTTC | CII-B |
| PaCII-B_R | AGGCGACGAGGAACTTGAC | CII-B |
| PaCIV-1_F | CAATTGGAGGGTTAAGTGGTG | CIV-1 |
| PaCIV-1_R | CCGCTAAACATTGCAAACAC | CIV-1 |
| PaCIV-2_F | CCAGAATCTGATTTAGATGAAGGA | CIV-2 |
| PaCIV-2_R | ACCTAAAGCAGGAGCAGCAA | CIV-2 |
| PaCIV-3_F | TGGGGAAAGAAAAGGAGCTT | CIV-3 |
| PaCIV-3_R | GTGGAAACCTGTCCCAAAAT | CIV-3 |
| PaG6P-DH_F | GACAGTATGGCAAGTCGCTGG | G6P-DH |
| PaG6P-DH_R | ACGGTACGCCATCCCACC | G6P-DH |
| Pa6PG-DH_F | TGCATGCCTCGTTATTGATGTC | 6PG-DH |
| Pa6PG-DH_R | GGAAATCAACAATACCATCGCTC | 6PG-DH |
| PaNADP-ME_F | GCAGCGACCAGGCCATCA | NADP-ME |
| PaNADP-ME_R | GGCAGCAGCAGAGGTGTAGATC | NADP-ME |
| PaNAD-ME_F | AGATCGAGGTTGCCGAGTGC | NAD-ME |
| PaNAD-ME_R | GGCAACAATGGCGCAGTATC | NAD-ME |
| PaCS-1_F | TACGCCGATGCCATTGCC | CS-1 |
| PaCS-1_R | TGTCGCTCAATGGCGCAC | CS-1 |
| PaCS-2_F | GCTGGCCAATGAGGACGAG | CS-2 |
| PaCS-2_R | CCAGTGTGCGAGACCACCG | CS-2 |
| PaCS-P_F | ATGTTCAAGCTCGTCAGCCAG | CS-P |
| PaCS-P _R | GCCTCGGTGAGACCATAGTACTG | CS-P |
| PaIDH1_F | TCCAGCGTGGCAAGCTCG | IDH1 |
| PaIDH1_R | AGTCCTCACGGCCAGTCTTGC | IDH1 |
| PaIDH2_F | TCCATCTTCGAGGCCGTCC | IDH2 |
| PaIDH2_R | CTTCTCGATGCGGTCGGC | IDH2 |
| PaFAS-1_F | TCTGCTCTGGCTGCTCTCGC | FAS-1 |
| PaFAS-1_R | ATGCGGCTGGGGTTGACG | FAS-1 |
| PaFAS-2_F | AGGCTCGCCAGAAGAAGGC | FAS-2 |
| PaFAS-2_R | AGTCTTCTTGTCTTCCGTGACGC | FAS-2 |
| PaSC-AD_F | GTACAATGCTGCTCGCAAGA | SC-AD |
| PaSC-AD_R | CTTCTCAGCCAGACCCTCAC | SC-AD |
| PaMC-AD_F | TTTGTGTCTACCGGGAAAGG | MC-AD |
| PaMC-AD_R | ATCATCCCTCTTGGCAAATG | MC-AD |
| PaLC-AD_F | GACAACGTCAAGGTCCCTGT | LC-AD |
| PaLC-AD_R | GCACTCCTCCGTTACGAGAC | LC-AD |
| PaAH-1_F | GATGCGTTTGCACAATTCAC | AH-1 |
| PaAH-1_R | CAATTTTGACTTCGGGGAGA | AH-1 |
| PaAH-2_F | GAGGACTGTGGGGAGACAGA | AH-2 |
| PaAH-2_R | TTAACCGCCTCCTCAACAAC | AH-2 |
| PaAH-3_F | CCTCATGGCGGACATAATCT | AH-3 |
| PaAH-3_R | TCTTCCCCGTCAATATCAGC | AH-3 |

**
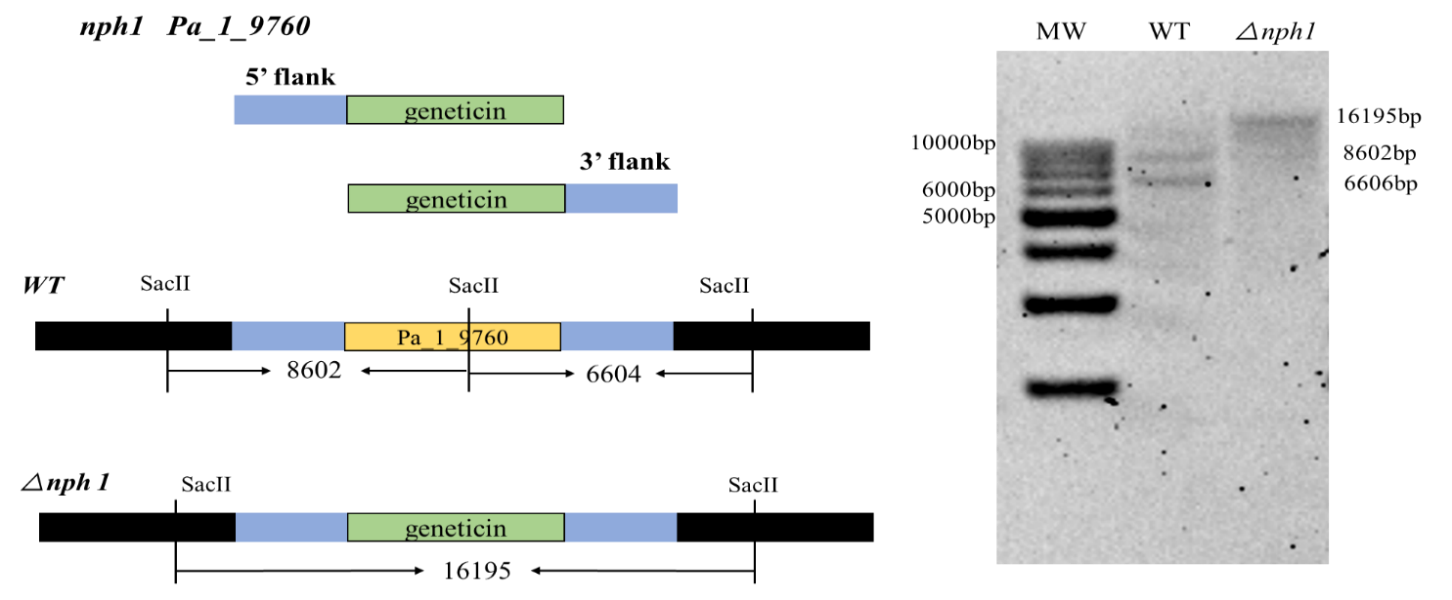
**


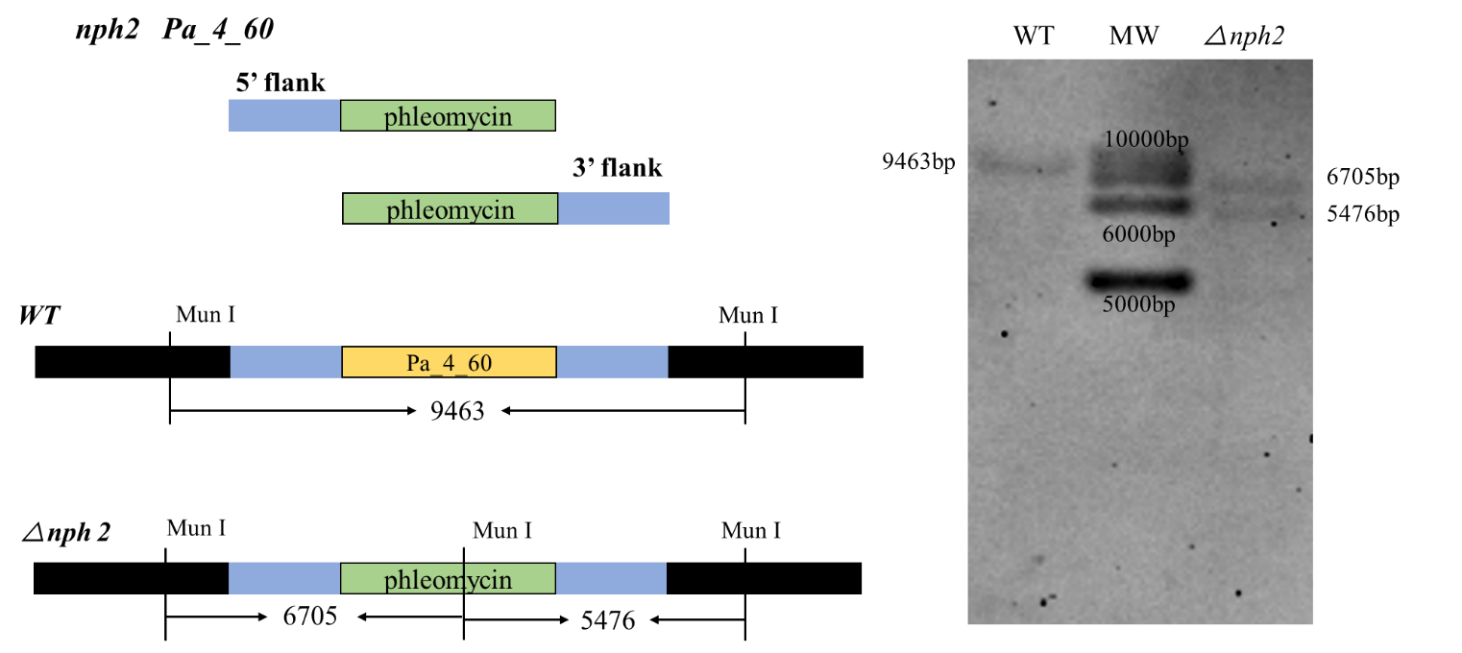


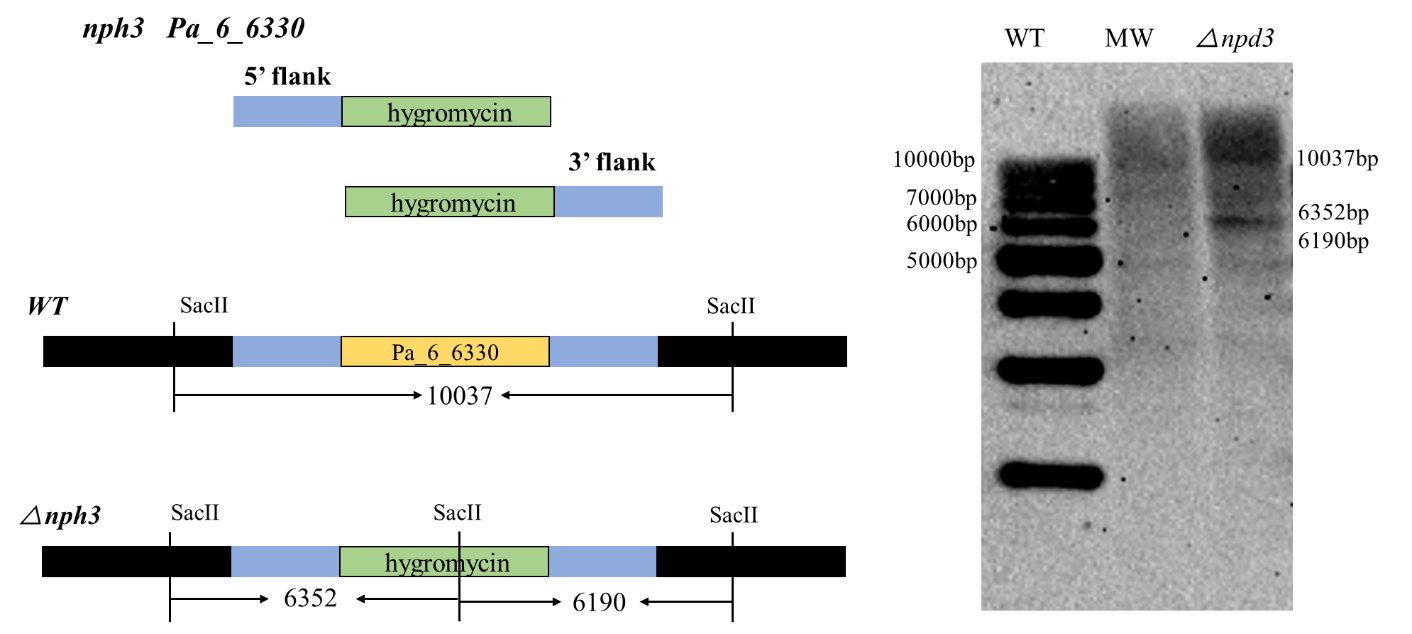


**Fig.S1.** Southern blotting analysis of the *^Δ^nph* mutants. DNA was isolated from wild-type *P. anserina* and from one or more purified transformants, and digested with appropriate restriction enzymes. The blots were probed with a sequence containing the relevant coding sequence (CDS) and its flanking regions. For each NADPH dehydrogenase gene, a restriction map of the wild-type and mutant locus is presented. The sizes of the expected fragments are indicated on the maps and are reported close to the corresponding fragment on the Southern blot.

**Fig.S2.** Linear correlation clarifying the mechanistic interactions between the number of fruiting bodies and the fluctuations in NADPH: NADP^+^ ratios between WT and mutant strains. Pearson correlation (r) = 0.961, N (i.e., WT, *nph1^△^*, *nph2^△^*, *nph3^△^*, *nph1^△^nph2^△^*, *nph1^△^nph3^△^*, *nph2^△^nph3^△^*, and *nph^△△△^*) = 8, DF = 6, *p*-value = 0.0001, and t-statistic = 8.47, where "t" is mathematically expressed as: $t=r x \frac{\sqrt{n-2}}{\sqrt{1-r^{2}}}$, DF = Degree of freedom. The Pearson correlation coefficient r = 0.961 (r^2^ ≈ 0.923) between the number of fruiting bodies and the NADPH: NADP⁺ ratio indicates a very strong **positive linear relationship**, and the **biological implication demonstrates that** higher intracellular reducing power (i.e., higher NADPH relative to NADP⁺) is strongly associated with increased fruiting body formation in *P. anserina*. In other words, assuming a linear model, such a high r^2^ value implies that **92.3% of the variation** in fruiting body number in *P. anserina* can be driven by NADPH: NADP⁺ dynamics.
